# Synthesis and conformational analysis of pyran inter-halide analogues of ᴅ-talose

**DOI:** 10.3762/bjoc.20.208

**Published:** 2024-09-27

**Authors:** Olivier Lessard, Mathilde Grosset-Magagne, Paul A Johnson, Denis Giguère

**Affiliations:** 1 Département de Chimie, 1045 av. De la Médecine, Université Laval, Québec City, Qc, G1V 0A6, PROTEO, Canada

**Keywords:** organofluorine, pyran inter-halide, solid-state conformation, solution-state conformation

## Abstract

In this work, we describe the synthesis of halogenated pyran analogues of ᴅ-talose using a halo-divergent strategy from known 1,6-anhydro-2,3-dideoxy-2,3-difluoro-β-ᴅ-mannopyranose. In solution and in the solid-state, all analogues adopt standard ^4^*C*_1_-like conformations despite 1,3-diaxial repulsion between the F2 and the C4 halogen. Moreover, the solid-state conformational analysis of halogenated pyrans reveals deviation in the intra-annular torsion angles arising from repulsion between the axial fluorine at C2 and the axial halogen at C4, which increases with the size of the halogen at C4 (F < Cl < Br < I). Crystal packing arrangements of pyran inter-halides show hydrogen bond acceptor and nonbonding interactions for the halogen at C4. Finally, density functional theory (DFT) calculations corroborate the preference of talose analogues to adopt a ^4^*C*_1_-like conformation and a natural bonding orbital (NBO) analysis demonstrates the effects of hyperconjugation from C–F antibonding orbitals.

## Introduction

Polyfluorinated pyran analogues of carbohydrates have attracted attention over the years. This class of glycomimetics has great biological potential with useful applications [1–7]. What about other halogens? Pyran inter-halide analogues of carbohydrates were rarely investigated as new tools in glycobiology [8]. This is surprising since the incorporation of halogens can improve cellular uptakes and enhance membrane binding and permeation [9–11]. In addition, halogen bonding is an important interaction in biological systems [12–17] and the beneficial effect of the chloro substituent has been reviewed recently [18].

**Figure 1:**
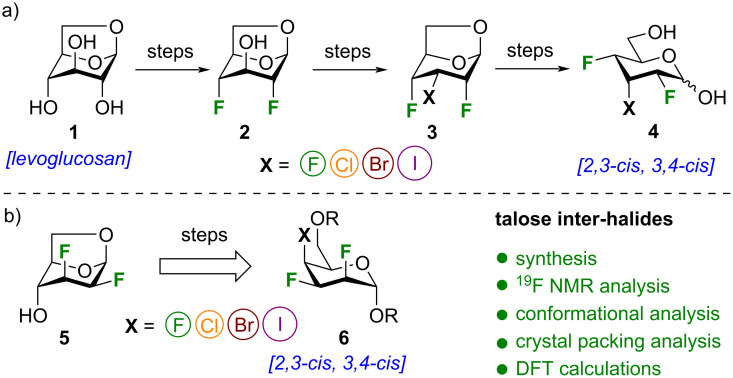
Synthesis of trihalogenated pyrans: a) Chiron approach to multivicinal inter-halide derived from allopyranoses; b) synthesis and conformational analysis of pyran inter-halide analogues of ᴅ-talose integrating the 2,3-*cis*, 3,4-*cis* relationship for the halogens (this work).

As a result, there is a lack of efficient synthetic strategies to access multivicinal inter-halide stereocenters (i.e., contiguous chiral halides: F, Cl, Br, I) [19]. Only a handful of natural product syntheses have been reported [20–21], despite the promising biological activity of these unique inter-halides [22]. For our part, we recently reported the synthesis of contiguous inter-halide-bearing stereocenters using a Chiron approach from levoglucosan **1** (Figure 1a) [23]. Allopyranose inter-halides **4** incorporating the 2,3-*cis*, 3,4-*cis* relationship for the halogens were prepared via intermediates **2** and **3** from levoglucosan (**1**). Compounds **4** were the starting point to complex 2,3,4-trihalohexanetriols and 2,3,4,5-tetrahalohexanediols. Conformational analysis and lipophilicities of the latter compounds were determined and trihalogenated alkanes were incorporated into piperidines of pitolisant [23]. This work was an extension of our synthetic routes to multivicinal organofluorines to unveil some of their unique properties [24–30], such as the solution-state conformation of diastereomeric polyfluorohexitols [31].

Herein, we report the synthesis of pyran inter-halide analogues of ᴅ-talopyranose **6**, integrating also the 2,3-*cis*, 3,4-*cis* relationship for the halogens, from known intermediate **5** (Figure 1b) [24]. The solution and the solid-state conformational analysis were supplemented with DFT calculations to understand key conformational drivers. This study adds more data to the field of nuclear magnetic resonance (NMR) spectroscopy of polyhalogenated molecules. It should be noted that the NMR predictions of such compounds remain very challenging [32].

## Results and Discussion

**Scheme 1:**
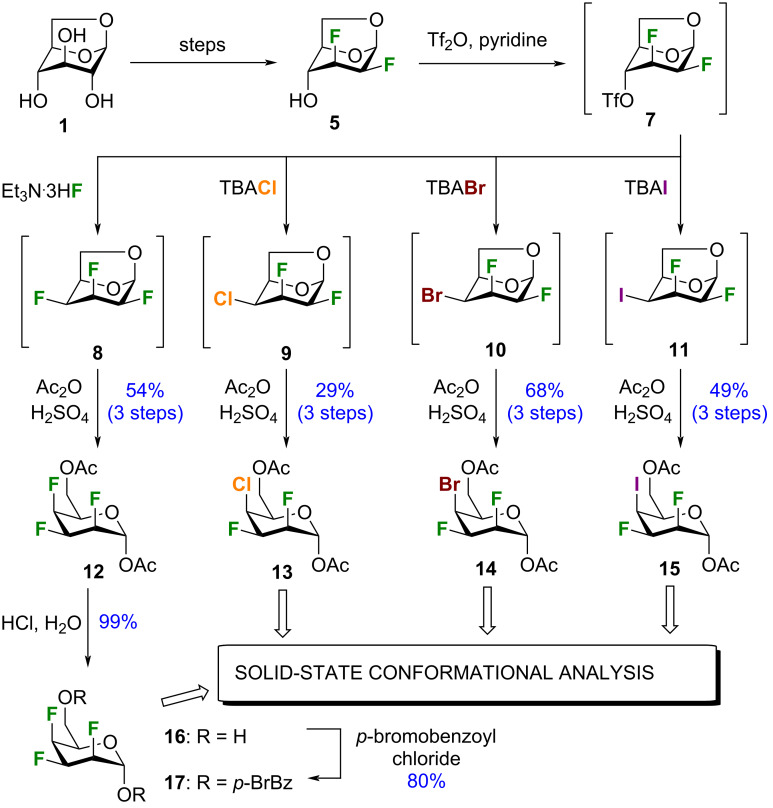
Synthesis of halogenated talopyranose analogues **13–15**, and **17** that include a 2,3-*cis*, 3,4-*cis* relationship for the halogens.

Our recent discovery that the nature of halogen atoms can have a large impact on conformation and lipophilicity motivated us to investigate novel pyran inter-halides [23]. We used a halo-divergent route starting from the known 1,6-anhydro-2,3-dideoxy-2,3-difluoro-β-ᴅ-mannopyranose (**5**) readily accessible form levoglucosan (**1**, Scheme 1) [24]. Activation of the C4 hydroxy group as triflate and direct treatment with a nucleophilic halogen furnished intermediates **8–11**. The latter compounds proved to be difficult to purify, therefore we were compelled to proceed directly to the next step. Cleavage of the 1,6-anhydro bridges was achieved under acetolysis conditions providing halogenated talopyranoses **12–15** in good yield over 3 steps as α anomers.

Luckily, inter-halides **13**–**15** were crystalline, allowing the absolute configuration to be confirmed by single-crystal diffraction analysis (see below) [33]. Unfortunately, trifluorinated analogue **12** was not crystalline. Thus, we removed the acetyl protecting groups and generated the corresponding *p*-bromobenzoate derivative **17** to obtain suitable crystalline material [24,34].

**Figure 2:**
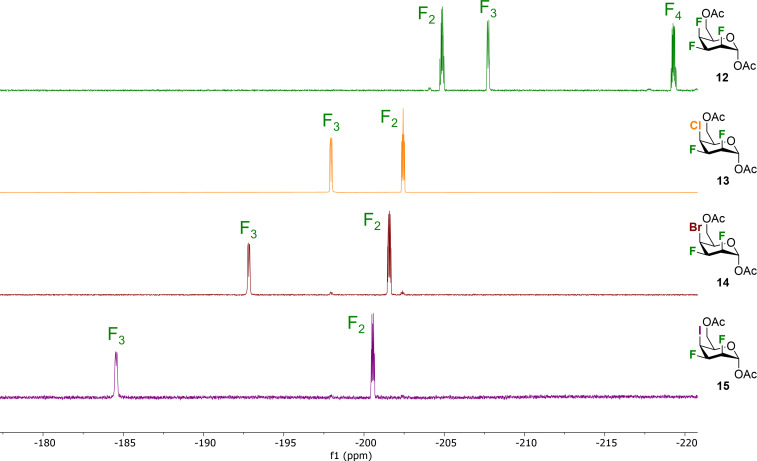
Direct comparison of ^19^F resonances of halogenated talose analogues **12**–**15** (^19^F NMR; 470 MHz, CDCl_3_).

In order to decipher the key physical properties of complex pyran inter-halides, we performed ^19^F NMR analysis of halogenated talose analogues **12**–**15** (Figure 2). First, all analogues adopt standard ^4^*C*_1_-like conformations. Comparison of the vicinal and geminal coupling constants for each organohalogen suggests that there is little change in the conformations (although there is an increasing chair distortion for larger halogens, see below). Because F3 is adjacent to the C4 halogen, ^19^F resonance of F3 occurs at lower field than F2 for analogues **13**–**15**. There is a strong increase in chemical shift of F3 depending on the incorporated halogen on the pyran core at C4: −208.33 ppm for **12** (fluorine), −197.95 ppm for **13** (chlorine), −192.80 ppm for **14** (bromine), and −184.56 ppm for **15** (iodine). Similarly, the increase in chemical shift of F2 is smaller as exemplified with an upfield shift of −205.46 ppm for **12** to −200.55 ppm for **15**. Talopyranose analogues **12–15** incorporate a 2,3-*cis*, 3,4-*cis* relationship for the halogens. We previously prepared a small set of trihalogenated allopyranose analogues that also included the 2,3-*cis*, 3,4-*cis* relationship for the halogens (Figure 1a) [23]. ^19^F NMR analysis of halogenated allopyranose analogues can be found in Supporting Information File 1.

**Figure 3:**
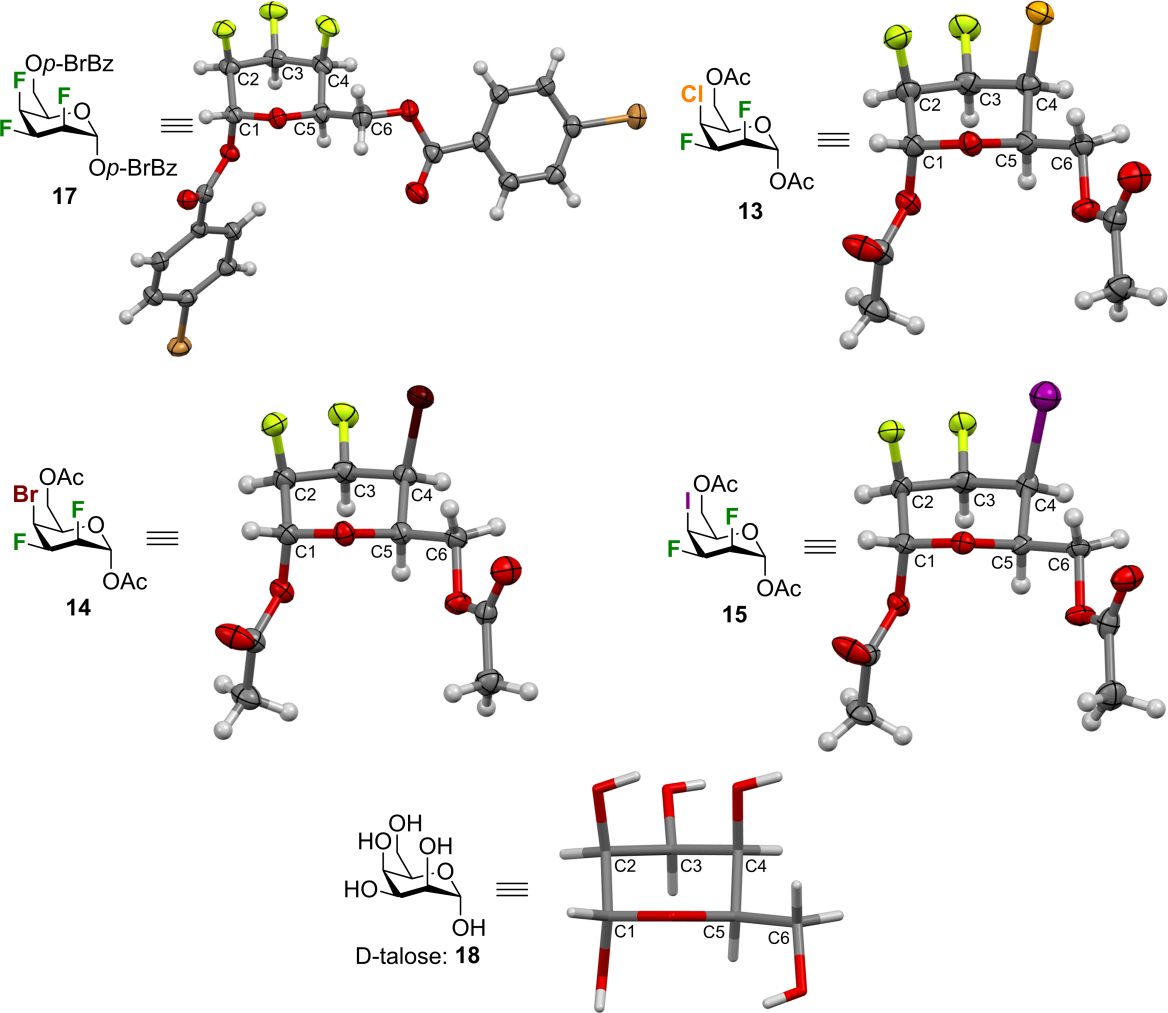
X-ray analysis of compound **13–15**, **17**, and α-ᴅ-talose **18**. ORTEP diagram showing 50% thermal ellipsoid probability (except for **18**): carbon (gray), oxygen (red), fluorine (green), chlorine (orange), bromine (dark red), iodine (purple), and hydrogen (white).

Our interest in the conformation of organohalogens motivated us to compare the solid-state conformation of halogenated pyrans **13**–**15**, and **17** [24] with α-ᴅ-talose **18** [35] (Figure 3). The crystallographic data and structural refinement details for the crystal structures can be found in Supporting Information File 1. As tosyl and benzoate groups are essential for the crystallinity of multivicinal organofluorines they influence the solid-state conformations [31,36–38]. Thus, information drawn from the crystallographic data of compound **17** might be influenced by benzoate groups. We included compound **17** in our comparative analysis in any case. All structures adopt a standard ^4^*C*_1_-like conformation in the solid-state. This conformation occurs despite 1,3-diaxial repulsion between the F2 and the C4 halogen. The 1,3-diaxial repulsion between 2 fluorine atoms have been reported in recent years [24,39], however, the 1,3-diaxial repulsion between fluorine and other halogens is quite uncommon [40–41]. As for the C5–C6 rotamer, all analogues exhibit a *gt* conformation except for trifluorinated **17**, which possesses a *tg* conformation. An axial substituent at C4 generally leads to a *gt* conformation [42–43], with some exceptions [24]. Bond distances, bond angles, torsion angles, and key interatomic distances are listed in Tables 1–4. It is important to point out that these results compare well with previous analysis of polyfluorinated carbohydrates [44–45].

The C–C bond lengths within the pyran rings of halogenated analogues are between 1.50 and 1.54 Å, which is similar to native talose (**18**, 1.52–1.53 Å) (Table 1, entries 1–4). However, all specified bond lengths within the pyran rings are shorter for halogenated analogues compared to α-ᴅ-talose, except for the C3–C4 bond of compound **15**. This can be explained by the adjacent repulsion between CF3 with the CI4 group. Next, it has been reported that the C1–O1 bond lengths are shorter than the O5–C1 for α anomers [42,46–47]. Talopyranose (**18**) follows this trend, but not the halogenated analogues (Table 1, entries 7 and 8). Also, the exocyclic C1–O1 bond lengths of compounds **13–15** and **17** are in average 0.033 Å longer than native talose (**18**). As expected, all the C–F bond lengths are shorter than the corresponding C–OH bond lengths (Table 1, entries 10–12) [48]. The C2–F2 bond lengths are in average 0.025 Å shorter than the C2–OH bond and the C3–F3 bond lengths are in average 0.027 Å shorter than the C3–OH bond. Similarly, for compound **13**–**15**, the C4–X bond lengths are longer than the C4–OH bond of native talose (1.43 Å): C4–Cl: 1.80 Å, C4–Br: 1.96 Å, and C4–I: 2.11 Å.

**Table 1:** Selected bond distances for compounds **13**–**15**, **17**, and **18**.

Entry	Bonds	Distances (Å)				

		Talose (**18**)^a^	**17**	**13**	**14**	**15**

1	C1–C2	1.5316	1.518(3)	1.526(2)	1.523(3)	1.53(1)
2	C2–C3	1.5234	1.506(3)	1.513(2)	1.508(3)	1.50(2)
3	C3–C4	1.5300	1.508(4)	1.522(2)	1.520(3)	1.54(2)
4	C4–C5	1.5325	1.524(3)	1.527(2)	1.523(3)	1.53(1)
5	C5–C6	1.5127	1.525(3)	1.507(2)	1.513(3)	1.50(1)
6	C5–O5	1.4489	1.432(3)	1.438(2)	1.435(2)	1.44(1)
7	O5–C1	1.4380	1.399(3)	1.403(2)	1.403(2)	1.40(1)
8	C1–O1	1.4028	1.442(3)	1.429(2)	1.431(2)	1.44(1)
9	O1–C(O)	na	1.361(3)	1.372(2)	1.374(3)	1.40(1)
10	C2–F2	1.4228^b^	1.393(3)	1.402(2)	1.407(2)	1.39(1)
11	C3–F3	1.4212^c^	1.397(3)	1.393(2)	1.396(2)	1.39(1)
12	C4–X4	1.4279^d^	1.395(3)	1.797(2)	1.956(2)	2.11(1)

^a^Reference [35]; ^b^C2–O2; ^c^C3–O3; ^d^C4–O4.

Table 2 shows the selected bond angles for compounds **13–15**, **17**, and **18**. All the bond angles of halogenated analogues are larger by 0.1–4.07° than talose (**18**). As such, the H2–C2–F2 bond angles are similar for compounds **13–15** and **17** (109.69–109.78°), but significantly larger than the H2–C2–O2 bond angle of talose (105.87°). Moreover, the H3–C3–F3 bond angles slightly decrease according to the nature of the atom at C4 (F: 107.97°; Cl: 107.67°; Br: 107.47°; I: 107.46°), as compared with talose (108.85°). As for the H4–C4–X4 bond angles, the angles are similar for talose and the trifluorinated analogues: 109.33° and 109.36°, respectively. However, there is a bond angle narrowing for the other analogues (Cl: 108.07°; Br: 107.94°; I: 107.78°). Finally, the angles involving the *exo*-anomeric oxygen (O1–C1–O5) are similar with a difference of about 1° between the larger (compound **17**) and smaller (compound **14**) angle.

**Table 2:** Selected bond angles for compounds **13**–**15**, **17**, and **18**.

Entry	Bonds	Angles (°)				

		Talose (**18**)^a^	**17**	**13**	**14**	**15**

1	C1–C2–C3	109.51	110.2(2)	111.9(1)	111.9(2)	111.9(9)
2	C2–C3–C4	110.43	113.7(2)	114.0(1)	114.5(2)	114.1(9)
3	C3–C4–C5	107.80	109.9(2)	108.3(1)	108.3(2)	107.9(8)
4	C4–C5–O5	109.92	111.8(2)	111.6(1)	112.2(1)	112.0(8)
5	C5–O5–C1	113.68	114.9(2)	114.7(1)	114.7(1)	113.9(8)
6	O5–C1–C2	110.29	113.0(2)	112.2(1)	112.3(2)	112.7(9)
7	O1–C1–O5	111.87	111.1(2)	111.3(1)	110.9(2)	111.4(8)
8	O1–C1–C2	107.98	105.5(2)	106.8(1)	106.9(2)	106.6(8)
9	C1–O1–C(O)	na	116.2(2)	115.4(1)	115.3(2)	115.2(8)
10	C1–C2–F2	109.78^b^	106.9(2)	105.4(1)	105.2(1)	104.6(8)
11	C3–C2–F2	112.49^c^	110.5(2)	110.4(1)	110.5(2)	110.9(9)
12	C2–C3–F3	107.54^d^	109.3(2)	109.5(1)	109.6(1)	109.9(9)
13	C4–C3–F3	113.36^e^	109.7(2)	110.1(1)	110.0(1)	110.1(8)
14	C3–C4–X4	108.18^f^	109.7(2)	112.1(1)	112.1(1)	111.9(7)
15	C5–C4–X4	111.14^g^	109.2(2)	112.1(1)	112.5(1)	113.5(7)
16	H2–C2–F2	105.87^h^	109.75	109.69	109.72	109.78
17	H3–C3–F3	108.85^i^	107.97	107.67	107.47	107.46
18	H4–C4–X4	109.33^j^	109.36	108.07	107.94	107.78

^a^Reference [35]; ^b^C1–C2–O2; ^c^C3–C2–O2; ^d^C2–C3–O3; ^e^C4–C3–O3; ^f^C3–C4–O4; ^g^C5–C4–O4; ^h^H2–C2–O2; ^i^H3–C3–O3; ^j^H4–C4–O4.

As stated above, all analogues exhibit a *gt* conformation except for compound **17**, which is a *tg* conformer. This information could also be extracted from Table 3 by looking at the O5–C5–C6–O6 torsion angles (Table 3, entry 1). Table 3 also highlights that there are significant intra-annular torsion angles for halogenated analogues. There are reductions in the C1–C2–C3–C4 torsion angles for the halogenated pyrans as compared to compound **18** (–56.58°) (Table 3, entry 4). The decrease depends on the size of the halogen at C4 (F: –49.4°; Cl: –46.9°; Br: –46.5°; I: –46°). There is also a reduction in the C2–C3–C4–C5 torsion angles of about 8.4° in average for compound **13–15** and **17** falling outside the range of an ideal pyran ring (Table 3, entry 5) [35]. However, the C4–C5–O5–C1 torsion angles are similar for all compounds except for compound **17** (Table 3, entry 7). The deviation in the intra-annular torsion angles clearly arise from repulsion of the axial fluorine at C2 and the axial halogen at C4 as exemplified with the H3–C3–C2–F2 and the X4–C4–C3–H3 torsion angles being smaller than the expected 180° (Table 3, entry 16 and 17). The 1,3-diaxial repulsion leads to a deviation from parallel alignment as shown in Table 4. The Newman projections of the halogenated analogues show deviations from parallel alignment for the C2–F and C4–X substituents of 12.08° for **17**, 17.08° for **13**, 18.18° for **14**, and 18.59° for **15** (talopyranose, **18**: 5.92°). This deviation is responsible for the distance between F2 and the halogen at C4. Table 5 highlights key interatomic distances for all analogues. As such, the F2∙∙∙X4 distance increases with the size of the C4 halogen (F < Cl < Br < I): 2.82 Å for **17**, 3.06 Å for **13**, 3.14 Å for **14**, and 3.23 Å for **15**. Another interesting feature can be drawn from Table 5. As such, intramolecular F2···F3, F2···X4, and F3···X4 contacts are smaller than the sum of the Van der Walls radii [49]. Taking together, all these data clearly demonstrate that the nature of one halogen can have an impact on the solid-state conformation of halogenated pyrans.

**Table 3:** Selected torsion angles for compounds **13–15**, **17**, and **18**.

Entry	Bonds	Torsion angles (°)				

		Talose **18**^a^	**17**	**13**	**14**	**15**

1	O5–C5–C6–O6	70.35	−178.3(2)	75.4(1)	74.9(2)	75(1)
2	O5–C1–O1–C(O)	na	94.7(2)	89.0(2)	88.7(2)	88(1)
3	C2–C1–O1–C(O)	na	−142.5(2)	−148.1(1)	−148.5(2)	−148.4(9)
4	C1–C2–C3–C4	−56.58	−49.4(3)	−46.9(2)	−46.5(2)	−46(1)
5	C2–C3–C4–C5	57.88	50.1(3)	50.0(2)	49.0(2)	49(1)
6	C3–C4–C5–O5	−58.49	−51.6(3)	−55.0(2)	−53.9(2)	−55(1)
7	C4–C5–O5–C1	60.88	56.4(2)	60.5(2)	59.8(2)	61(1)
8	C5–O5–C1–C2	−58.73	−56.1(2)	−55.5(2)	−55.3(2)	−57(1)
9	O5–C1–C2–C3	55.31	51.0(3)	47.6(2)	47.6(2)	48(1)
10	O5–C1–C2–F2	−68.66^b^	−69.0(2)	−72.4(1)	−72.4(2)	−72(1)
11	C1–C2–C3–F3	179.26^c^	−172.3(2)	−170.7(1)	−170.7(1)	−170.6(8)
12	F2–C2–C3–C4	65.78^d^	68.5(3)	70.2(2)	70.4(2)	70(1)
13	F3–C3–C4–C5	178.62^e^	172.8(2)	173.5(1)	173.0(1)	173.1(8)
14	C2–C3–C4–X4	−62.40^f^	−70.0(3)	−74.2(2)	−75.6(2)	−77(1)
15	X4–C4–C5–O5	59.91^g^	68.8(2)	69.2(1)	70.5(2)	70.0(9)
16	X4–C4–C3–H3	179.05^h^	170.2	166.4	165.0	164.4
17	H3–C3–C2–F2	−175.98^i^	−171.7	−170.4	−170.3	−170.9

^a^Reference [35]; ^b^O5–C1–C2–O2; ^c^C1–C2–C3–O3; ^d^O2–C2–C3–C4; ^e^O3–C3–C4–C5; ^f^C2–C3–C4–O4; ^g^O4–C4–C5–O5; ^h^O4–C4–C3–H3; ^i^H3–C3–C2–O2.

**Table 4:** 1,3-Diaxial repulsion between C2–F and C4–X bonds for **13–15** and **17**.^a^

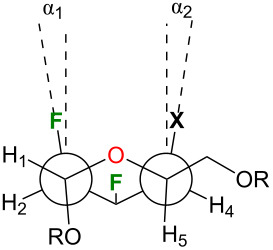					

Entry	Angles (°)	**17** (X = F, R = *p*-BrBz)	**13** (X = Cl, R = Ac)	**14** (X = Br, R = Ac)	**15** (X = I, R = Ac)

1	α_1_	5.99	6.77	7.05	7.06
2	α_2_	6.09	10.31	11.13	11.53
3	α_1_ + α_2_	12.08	17.08	18.18	18.59

^a^For α-talopyranose **18**: α_1_ = 5.58°, α_2_ = 0.34°, and α_1_ + α_2_ = 5.92° (reference [35]).

**Table 5:** Key interatomic distances (intramolecular) for **13–15**, **17**, and **18**.

Entry	Atoms	Distances (Å)				

		Talose (**18**)^a^	**17**	**13**	**14**	**15**

1	O1∙∙∙F2	3.6141^b^	3.560(2)	3.552(2)	3.557(2)	3.55(1)
2	O1∙∙∙F3	4.1700^c^	4.194(2)	4.288(2)	4.285(2)	4.28(1)
3	F2∙∙∙F3	2.8154^d^	2.732(3)	2.735(2)	2.741(2)	2.746(9)
4	F2∙∙∙X4	2.6546^e^	2.817(2)	3.056(1)	3.143(1)	3.228(6)
5	F3∙∙∙X4	2.8517^f^	2.714(2)	2.968(1)	3.052(1)	3.160(6)

^a^Reference [35]; ^b^O1∙∙∙O2; ^c^O1∙∙∙O3; ^d^O2∙∙∙O3; ^e^O2∙∙∙O4; ^f^O3∙∙∙O4.

We also evaluated the Cremer–Pople ring puckering parameters (Table 6) [50]. For pyranoid rings, these parameters take the form of a spherical polar coordinate set, *Q*, θ, and φ, which define the point P, on a sphere of radius *Q* [51]. The smaller puckering amplitude (*Q*) values for the halogenated analogues indicate a flattened ring in comparison to the non-halogenated compound. The puckering amplitude for an ideal cyclohexane chair, with C–C bond lengths of 1.54 Å, is 0.63 Å [50]. The azimuthal angle (θ) represents the distortion of the ring. For pyranose rings, an azimuthal angle of θ = 0° represents a perfect ^4^*C*_1_ chair, and an angle of θ = 180° is the ^1^*C*_4_ chair. The distortion of the chair conformation increases with the size of the halogen at C4. Surprisingly, the trifluorinated analogue is less distorted than the non-halogenated talopyranose. The meridian angle (φ) indicates the nature of the distortion. The distortion of the trifluorinated analogue is in a direction somewhat between an *E*_5_ conformation (φ ≈ 300°) and a ^O^*H*_5_ conformation (φ ≈ 330°). The other trihalogenated analogues are distorted towards an *E*_5_ conformation (φ ≈ 300°). The distortion of the non-halogenated talopyranose is in the direction on an ^4^*E* conformation (φ ≈ 240°).

**Table 6:** Cremer–Pople ring puckering amplitudes (*Q*), theta (θ) and phi (φ) parameters.

	Talose (**18**)	**17**	**13**	**14**	**15**

*Q* (Å)	0.588	0.514	0.521	0.514	0.523
θ (°)	2.976	1.724	6.936	6.439	7.248
φ (°)	233.600	315.204	299.454	305.438	307.711

**Figure 4:**
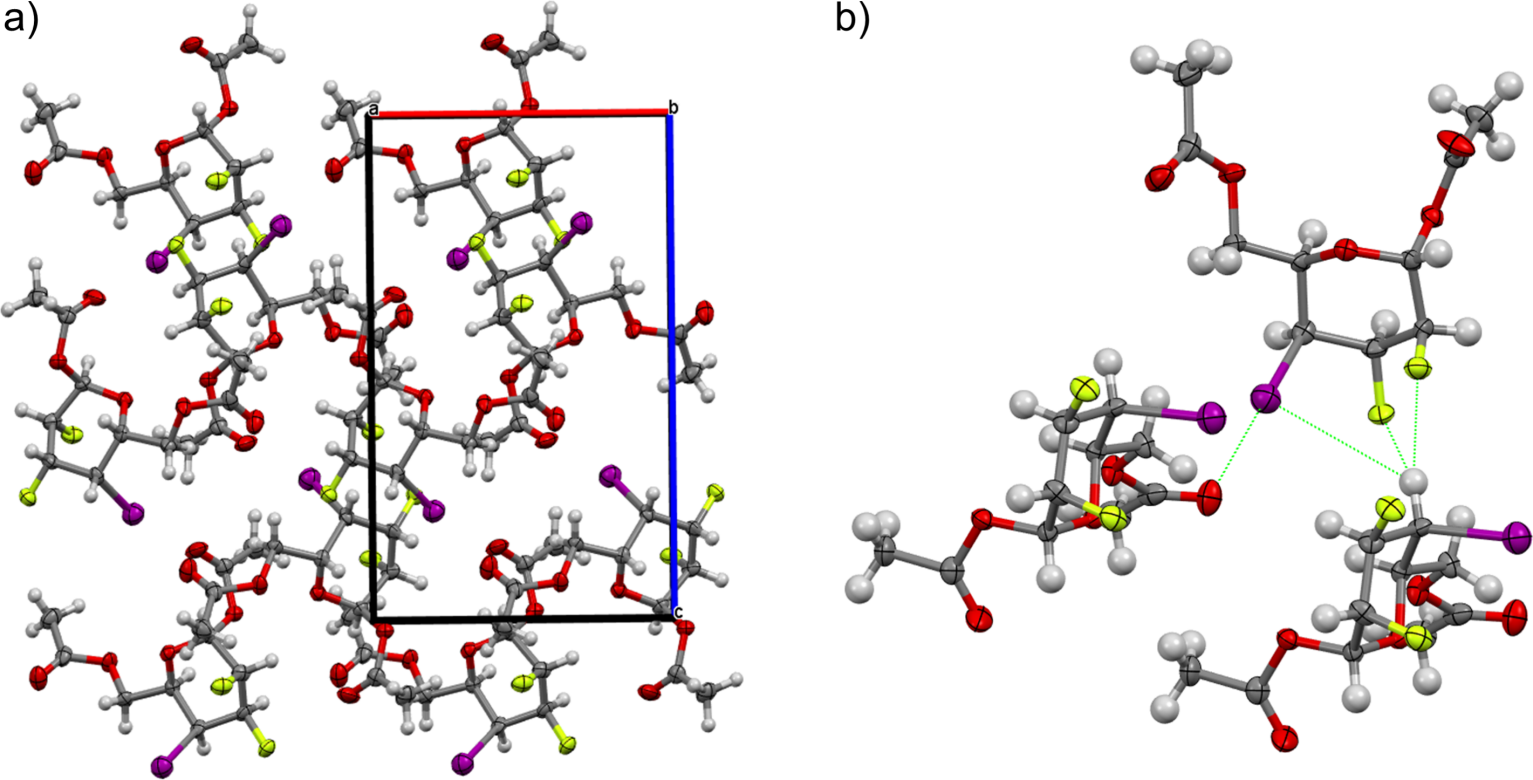
Packing arrangement of compound compound **15**; a) View down the *b* axis; b) proposed intermolecular interactions involving halogens. ORTEP diagram showing 50% thermal ellipsoid probability: carbon (gray), oxygen (red), fluorine (green), iodine (purple), and hydrogen (white).

The solid-state conformation of each of the pyran inter-halides **13–15** is unique. One would expect that compounds **13–15** would have distinct crystal packing arrangements based on the nature of the halogen. On the contrary, all analogues adopt a similar stacking pattern. Figure 4 shows the packing arrangement for compound **15** and the crystal packing of compound **13** and **14** can be found in the Supporting Information File 1. The halogens are on the same side of the pyran ring, thus increasing the overall molecular dipole moment (see Supporting Information File 1). This allows intermolecular C–X···H–C interactions responsible, in part, for the solid-state ordering [52]. Individual pyrans stack on top of one another in a manner consistent with electrostatic interactions with halogens facing H3, H4, and H5 (Figure 4a). As such, some intermolecular H···X bond distances and angles for compound **13**–**15** are listed in Table 7. Solid-state intermolecular interactions involving fluorine atoms have been well documented over the years for carbohydrate analogues [24,44,53] or other organofluorines [54]. In our case, there is a number of C–F···H–C interactions with F2 and H4 (**13**: *d* = 2.271 Å, **14**: *d* = 2.270 Å, and **15**: *d* = 2.356 Å) and with F3 and H4 (**13**: *d* = 2.867 Å, **14**: *d* = 2.849 Å, and **15**: *d* = 2.886 Å).

**Table 7:** Intermolecular X···H bond distances and angles for compound **13**–**15**.

Entry	Compound	D–H···A	*d*(D–H) (Å)	*d*(H···A) (Å)	*d*(D···A) (Å)	*a*(D–H–A) (°)	*a*(C–X–H) (°)

1	**13**	F2···H3	0.980	3.704	3.826	89.69	na
2		F2···H4	0.980	2.271	3.222	163.14	na
3		F3···H3	0.980	4.239	4.561	103.01	na
4		F3···H4	0.980	2.867	3.614	133.69	na
5		F3···H5	0.980	3.074	3.490	107.03	na
6		Cl4···H3	0.980	3.437	4.232	139.73	133.71
7		Cl4···H4	0.980	3.625	4.298	128.02	96.94
8		Cl4···H5	0.980	3.953	4.624	128.19	103.45

9	**14**	F2···H3	1.000	3.771	3.869	88.12	na
10		F2···H4	1.000	2.270	3.233	161.19	na
11		F3···H3	1.000	4.290	4.599	101.79	na
12		F3···H4	1.000	2.849	3.626	135.07	na
13		F3···H5	1.000	3.130	3.533	105.61	na
14		Br4···H3	1.000	3.316	4.132	139.90	133.33
15		Br4···H4	1.000	3.526	4.213	127.67	95.15
16		Br4···H5	1.000	3.887	4.564	127.36	102.99

17	**15**	F2···H3	0.980	3.867	3.950	87.64	na
18		F2···H4	0.981	2.356	3.293	159.70	na
19		F3···H3	0.980	4.382	4.673	101.24	na
20		F3···H4	0.981	2.886	3.668	137.42	na
21		F3···H5	0.980	3.248	3.612	103.92	na
22		I4···H3	0.980	3.285	4.086	140.25	132.28
23		I4···H4	0.981	3.521	4.192	127.59	93.77
24		I4···H5	0.980	3.905	4.559	126.76	102.70

Does the halogen at C4 contribute to the stabilization within the crystal lattice? To answer this question, we have to look at the behavior of halogens as hydrogen bond acceptors (X···H) and nonbonding interactions (X···O/N/S). For C–X, a σ-hole arises when a valence electron is pulled into the σ-molecular orbital resulting in an electropositive crown and a flattening of the atomic radius, that accounts for the directionality of the interactions [55–56]. Thus, the halogen has an amphoteric character with an electropositive halogen bond ability along the σ-hole (C–X···O/N/S, *a* ≈ 180°) and an electronegative hydrogen bond acceptor perpendicular to the C–X bond (C–X···H, *a* ≈ 90°) [57–60]. Such halogen bonds have been detrimental in the understanding interactions of organic halogens in biological systems [61–65]. In our case, for compound **15**, I4 interacts with H4 (*d* = 3.521 Å, *a* = 93.77°) and I4 also interact with the oxygen of the carbonyl of the acetate at C6 (I4···O, *d* = 3.147 Å; *a* = 179.70°) (Figure 4b and Table 7). This result is in line with hydrogen (C–I···H) and halogen (C–I···O) interactions that show remarkable differences in term of geometrical features [66]. It is important to point out that similar interactions are also present in the packing of compound **13** (Cl4···H4 (*d* = 3.625 Å, *a* = 96.94°) and Cl4···O (*d* = 3.203 Å, *a* = 174.03°)) and compound **14** (Br4···H4 (*d* = 3.526 Å, *a* = 95.15°) and Br4···O (*d* = 3.143 Å, *a* = 177.49°)) (Table 7) (and see Supporting Information File 1). To the best of our knowledge, this is the first application of halogen bonding in the context of solid-state ordering of pyran inter-halides.

Our interest in the synthesis and conformation of multivicinal inter-halides motivated us to use density functional theory (DFT) calculations to understand the preference of talose analogues to adopt ^4^*C*_1_-like conformations. DFT computations were performed using Gaussian 16 revision B.01 [67] with the CAM-B3LYP [68–70] functional and the Def2TZVP basis set [71], which includes effective core potentials for iodine. Empirical dispersion was accounted with Grimme’s D3 [72–73] correction including Becke–Johnson damping [74]. Computations were performed both in the gas phase (i.e., individual molecules with thermal corrections at 298.15 K based on ideal gas assumptions) and in a chloroform solution, using the polarizable continuum model (PCM) [75]. A natural bonding orbital (NBO) analysis was performed to study the effects of hyperconjugation from C–F antibonding orbitals [76].

First, dipole moments, enthalpy and Gibbs free energy differences between ^1^*C*_4_ and ^4^*C*_1_ chair structures are shown in Table 8. In the gas phase, there is little difference in enthalpy between the two structures. For Cl, Br, and I, the ^1^*C*_4_ structures are predicted to have slightly lower enthalpies. However, as these differences are much smaller than 1 kcal/mol, one should conclude that the structures are nearly degenerate. In solution, the picture is much clearer: the ^4^*C*_1_ structure is always lower in enthalpy and Gibbs free energy, which corresponds with the experimental measurements. One can see that the gap between the two structures tends to decrease as the halogen becomes larger (the minor exception being bromine). Dipole moments for the ^4^*C*_1_ structures are 2 Debye larger than for ^1^*C*_4_ structures, making them substantially more stable in chloroform.

**Table 8:** Dipole moments (Debye), enthalpy and Gibbs free energy differences (kcal/mol) computed (CAM-B3LYP-D3BJ/Def2TZVP) for ^1^*C*_4_ and ^4^*C*_1_ chair structures in the gas phase and in chloroform (PCM). Thermal corrections are reported at 298.15 K.

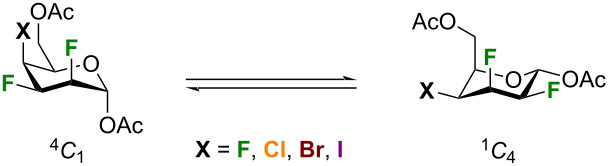									

		Gas phase				Chloroform			

Entry	X	μ ^1^*C*_4_	μ ^4^*C*_1_	Δ*H*	Δ*G*	μ ^1^*C*_4_	μ ^4^*C*_1_	Δ*H*	Δ*G*

1	F	4.55	6.48	0.98	2.51	5.95	8.09	2.47	3.90
2	Cl	4.45	6.34	−0.03	1.31	5.93	7.93	1.29	2.21
3	Br	4.40	6.30	−0.13	1.18	5.89	7.94	1.19	2.55
4	I	4.32	6.07	−0.03	1.17	5.78	7.64	1.04	1.86

Both chair structures have multiple C–F bonds in gauche arrangements which are stable due to hyperconjugation: there is donation from C–H bonding orbitals to C–F (or C–X) antibonding orbitals that are aligned with one another. The principal difference between the two structures is that in the ^1^*C*_4_ structure there are two such interactions whereas in the ^4^*C*_1_ structure there are three. One can see that in the ^4^*C*_1_ structure the C5–H5 bond will donate to the antibonding orbital of the C4–X4 bond, and the C3–H3 bond will donate to the antibonding orbitals of the C4–X4 and the C2–F2 bonds. In the ^1^*C*_4_ structure, the C4–H4 and C2–H2 bonds will both donate to the antibonding orbital of the C3–F3 bond. These effects can be shown with an NBO analysis: the Kohn–Sham orbitals produced from DFT are localized to describe the system as one dominant resonance structure. As the Fock matrix is not diagonal in terms of the NBOs, coupling between orbitals can be quantified with second order perturbation theory. These couplings represent donation from an occupied NBO to an unoccupied NBO that would stabilize the system. The results are presented in Table 9. In both cases, there is also donation from the halogen lone pairs to C–H and C–C antibonding orbitals, but as these effects were near equivalent in both chair structures, they are omitted.

**Table 9:** Second order perturbation theory energies of the Fock matrix in the NBO basis for ^4^*C*_1_ and ^1^*C*_4_ structures, from CAM-B3LYP-D3BJ/Def2TZVP results. Only results from CHCl_3_ solvation (PCM) are reported as gas phase results showed no qualitative difference. Entries marked – are below the 0.50 kcal/mol threshold.

			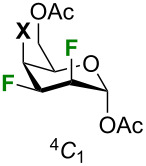				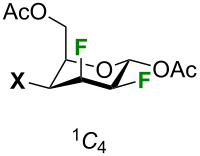			
				
Entry	Donor σ	Acceptor σ*	X = F	X = Cl	X = Br	X = I	X = F	X = Cl	X = Br	X = I

1	C5–H5	C4–X4	5.07	6.58	7.46	7.83	–	–	–	–
2	C4–H4	C4–X4	1.05	0.88	0.97	0.75	1.10	0.80	0.81	0.56
3	C4–H4	C3–F3	–	–	–	–	5.18	5.53	5.67	5.88
4	C3–H3	C4–X4	5.36	6.59	7.46	7.71	0.61	–	–	–
5	C3–H3	C3–F3	1.05	1.03	1.02	1.02	1.00	1.02	1.02	1.05
6	C3–H3	C2–F2	5.00	5.18	5.20	5.22	0.58	0.59	0.59	0.61
7	C2–H2	C3–F3	0.50	0.50	0.50	0.52	4.63	4.61	4.58	4.58
8	C2–H2	C2–F2	1.01	0.99	0.99	0.99	1.01	1.00	1.00	1.01
9	C1–H1	C2–F2	1.10	1.21	1.22	1.26	0.53	0.56	0.57	0.56

## Conclusion

We described the synthesis and conformational analysis of halogenated pyran analogues of ᴅ-talose. All analogues adopt standard ^4^*C*_1_-like conformations both in solution and in the solid-state. The conformations were corroborated using DFT calculations by looking at the energy, enthalpy and Gibbs’ free energy differences between ^1^*C*_4_ and ^4^*C*_1_ chair structures. Crystallographic data of halogenated analogues shows intra-annular torsion angles demonstrated with the increasing distance between F2∙∙∙X4 in relation with the nature of the halogen at C4: F (*d* = 2.82 Å) < Cl (*d* = 3.06 Å) < Br (*d* = 3.14 Å) < I (*d* = 3.23 Å). Moreover, the Cremer–Pople ring puckering parameters show suitable differences in the distortion of the chair conformations. Crystal packing arrangements showed that the halogen at C4 contributed in the nonbonding (along the σ-hole) and hydrogen bond (perpendicular to the C–X bond) interactions. Finally, this study should be of general interest in the understanding of weak interactions that are now important to so many areas of chemistry, such as crystal engineering and supramolecular chemistry.

## Supporting Information

File 1 Experimental and analytical data, crystal structure determination and NMR spectra.

## Data Availability

All data that supports the findings of this study is available in the published article and/or the supporting information to this article.
