# Supplementary material for: Synthesis and conformational analysis of pyran inter-halide analogues of ᴅ-talose
Source: Beilstein J Org Chem. 2024 Sep 27;20:2442–54. doi: 10.3762/bjoc.20.208 (PMC11443651; doi:10.3762/bjoc.20.208)
Supplement: File 1 — Experimental and analytical data, crystal structure determination and NMR spectra. [file Beilstein_J_Org_Chem-20-2442-s001.pdf]

BEILSTEIN JOURNAL OF ORGANIC CHEMISTRY

Supporting Information

for

Synthesis and conformational analysis of pyran inter-halide analogues of D-talose

Olivier Lessard, Mathilde Grosset-Magagne, Paul A. Johnson and Denis Giguère

Beilstein J. Org. Chem. **2024,** *20,* 2442–2454. doi:10.3762/bjoc.20.208

Experimental and analytical data, crystal structure determination and NMR spectra

License and Terms: This is a supporting information file under the terms of the Creative Commons Attribution License (https://creativecommons.org/licenses/by/4.0). Please note that the reuse, redistribution and reproduction in particular requires that the author(s) and source are credited and that individual graphics may be subject to special legal provisions.
The license is subject to the *Beilstein Journal of Organic Chemistry* terms and conditions: (https://www.beilstein-journals.org/bjoc/terms)

Table of contents

I. Experimental section……………………………………………………………..S2

II. Comparison of ^{19}F resonances of halogenated talose and allose analogues……....S6

III. Crystal structure determination………………………………………………..S7

IV. Crystal packing…………………………………………………………………S11

V. Density functional theory calculations…………………………………………S13

VI. NMR spectra of compounds……………………………………………………S30

VII. References………………………………………………………………………S38

I. Experimental section

General methods

All reactions were carried out under an argon atmosphere with dry solvents under anhydrous conditions, unless otherwise noted. Dry dichloromethane (CH_2Cl_2) was obtained by passing commercially available pre-dried, oxygen-free formulations through activated alumina columns using a Vacuum Atmospheres Inc. Solvent Purification System. Yields refer to chromatographically and spectroscopically (^{1}H NMR) homogeneous materials, unless otherwise stated. Reagents were purchased at the highest commercial quality available and used without further purification, unless otherwise stated. Reactions were monitored by thin-layer chromatography (TLC) carried out on 0.25 mm E. Merck silica gel plates (60F-254) using UV light as visualizing agent and charring with a $KMnO_4$ solution (1.5 g of $KMnO_4$, 10 g K_2CO_3, and 1.25 mL 10% NaOH in 200 mL of water), or a phenol solution (3 g phenol, 5 mL conc. H_2SO_4 in 95 mL of EtOH), followed by heating with a heatgun as developing agents. SiliaFlash® P60 (particle size 40–63 mm, 230–400 mesh) was used for flash column chromatography. NMR spectra were recorded on an Agilent DD2 spectrometer (at 500 MHz for ^{1}H, 470 MHz for ^{19}F, and 126 MHz for ^{13}C) and calibrated using residual undeuterated solvent peaks ($CDCl_3$ ^{1}H δ = 7.26 ppm, ^{13}C δ = 77.16 ppm; acetone-d_6: ^{1}H δ = 2.05 ppm, ^{13}C δ = 29.84 ppm) as an internal reference. Coupling constants (*J*) are reported in Hertz (Hz), and the following abbreviations were used to designate multiplicities: s = singlet, d = doublet, t = triplet, q= quartet, p = quintet, m = multiplet, br = broad. Assignments of NMR signals were made by homonuclear (COSY) and heteronuclear (HSQC, HMBC, and ^{19}F gc2HSQC) two-dimensional correlation spectroscopy. Infrared (IR) spectra were recorded using an ABB Bomem MB-Series Arid Zone FTIR MB-155 Spectrometer, with a ZnSe crystal plate. The absorptions are given in wavenumbers (cm^{-1}). High-resolution mass spectra (HRMS) were measured with an Agilent 6210 LC Time of Flight mass spectrometer in electrospray mode (ESI). Either protonated molecular ions $[M + nH]^{n+}$, sodium adducts $[M + Na]^+$, ammonium adducts $[M + NH_4]^+$ or deprotonated molecular ions $[M - nH]^{n-}$ were used for empirical formula confirmation. Optical rotations were recorded on a JASCO DIP-360 digital polarimeter at 589 nm and are reported in units of 10^{-1} (deg cm^2 g^{-1}). Melting points were measured on a Stanford Research System OptiMelt MPA100 151 automated melting point apparatus.

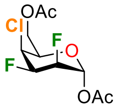

13

1,6-Di-*O*-acetyl-4-chloro-2,3,4-trideoxy-2,3-difluoro-α-D-talopyranose (13). In a manner similar to Denavit et al. [1], to a stirred solution of 1,6-anhydro-2,3-dideoxy-2,3-difluoro-β-D-mannopyranose **5** (45.4 mg, 0.2733 mmol) in CH_2Cl_2 (1.4 mL, 0.2 M) at 0 °C, were added pyridine (66.4 μL, 0.8199 mmol, 3 equiv) and Tf_2O (69.0 μL, 0.4099 mmol, 1.5 equiv). The mixture was stirred at room temperature for 30 min and then quenched with water (3 mL). The mixture

was extracted with CH_2Cl_2 (3 × 5 mL), and the combined organic phases were successively washed with an aqueous 1 M HCl solution (5 mL) and brine (5 mL). The organic solution was dried over $MgSO_4$, filtered, and concentrated under reduced pressure. The crude triflate **7** was used for the next step without further purification. To a stirred solution of the crude triflate **7** in anhydrous acetonitrile (2.7 mL, 0.1 M) was added tetrabutylammonium chloride (227.9 mg, 0.8199 mmol, 3 equiv). The mixture was stirred at 65 °C for 6 days. The volatiles were removed under reduced pressure and the crude was dissolved in CH_2Cl_2 (2.7 mL, 0.1 M). The mixture cooled to 0 °C and Ac_2O (0.76 mL, 8.199 mmol, 30 equiv) and H_2SO_4 (0.15 mL, 2.733 mmol, 10 equiv) were added. The mixture was stirred at room temperature for 18 h, then cooled to 0 °C. Sodium acetate (450 mg, 5.466 mmol, 20 equiv) was added and the mixture was stirred for an additional 20 min. Water (3 mL) was added and the mixture was extracted with CH_2Cl_2 (3 × 5 mL). The combined organic phases were successively washed with a saturated aqueous $NaHCO_3$ solution (5 mL) and brine (5 mL). The organic solution was dried over $MgSO_4$, filtered, and concentrated under reduced pressure. The crude residue was purified by flash column chromatography (silica gel, EtOAc/Hexanes, 1:4 → 1:1) to give **13** (α/β, >20:1) as a white solid (22.6 mg, 0.07884 mmol, 29% over 3 steps). The resulting product was recrystallized from acetone/heptane to give colorless crystals. R_f = 0.35 (silica, EtOAc/hexanes 1:1); m.p. = 153 – 162 °C; $[\alpha]_D^{25}$ = 89.5 (*c* 0.5, $CHCl_3$); IR (ATR, diamond crystal) ν 3015, 2932, 1759, 1732, 1242, 1024, 978 cm^{-1}; ^1H NMR (500 MHz, $CDCl_3$) δ 6.37 (ddd, $^3J_{H1–F2}$ = 8.0 Hz, $^3J_{H1–H2}$ = 5.5 Hz, $^4J_{H1–F3}$ = 1.9 Hz, 1H, H1), 4.95 (ddt, $^2J_{H3–F3}$ = 43.2 Hz, $^3J_{H3–F2}$ = 26.4 Hz, $^3J_{H3–H2}$ = $^3J_{H3–H4}$ = 3.6 Hz, 1H, H3), 4.74 (ddddd, $^2J_{H2–F2}$ = 48.7 Hz, $^3J_{H2–H1}$ = 5.5 Hz, $^3J_{H2–H3}$ = 3.5 Hz, $^3J_{H2–F3}$ = 2.0 Hz, $^4J_{H2–H4}$ = 1.0 Hz, 1H, H2), 4.44 (ddd, $^3J_{H4–F3}$ = 4.3 Hz, $^3J_{H4–F3}$ = 3.6 Hz, $^4J_{H4–H2}$ = 1.0 Hz, 1H, H4), 4.41 – 4.29 (m, 3H, H5, H6a, H6b), 2.14 (s, 3H, COC*H*$_3$), 2.09 (s, 3H, COC*H*$_3$) ppm; ^{13}C {^1H} NMR (126 MHz, $CDCl_3$) δ 170.65 (1C, *C*OCH$_3$), 167.80 (1C, *C*OCH$_3$), 91.20 (dd, $^1J_{C1–F2}$ = 32.3 Hz, $^3J_{C1–F3}$ = 6.9 Hz, 1C, C1), 84.24 (dd, $^1J_{C2–F2}$ = 190.9 Hz, $^2J_{C2–F3}$ = 17.0 Hz, 1C, C2), 83.88 (dd, $^1J_{C3–F3}$ = 197.7 Hz, $^2J_{C3–F2}$ = 16.0 Hz, 1C, C3), 69.28 (d, $^3J_{C5–F3}$ = 4.2 Hz, 1C, C5), 63.08 (d, $^4J_{C6–F3}$ = 3.3 Hz, C6), 54.19 (d, $^2J_{C4–F3}$ = 18.3 Hz, 1C, C4), 20.89 (1C, CO*C*H$_3$), 20.87 (1C, CO*C*H$_3$) ppm; ^{19}F NMR (470 MHz, $CDCl_3$) δ -197.95 (ddq, $^2J_{F3–H3}$ = 43.2 Hz, $^3J_{F3–F2}$ = 14.8 Hz, $^3J_{F3–H4}$ = 4.3 Hz, $^3J_{F3–H2}$ = 2.0 Hz, $^4J_{F3–H1}$ = 1.9 Hz, 1F, F3), -202.42 (dddd, $^2J_{F2–H3}$ = 49.5 Hz, $^3J_{F2–H3}$ = 26.5 Hz, $^3J_{F2–F3}$ = 14.8 Hz, $^3J_{F2–H1}$ = 8.5 Hz, 1F, F2) ppm; HRMS calcd for $C_{10}H_{17}ClF_2NO_5^+$ $[M + NH_4]^+$ 304.0758 found 304.0759.

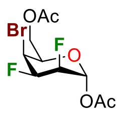

14

1,6-Di-*O*-acetyl-4-bromo-2,3,4-trideoxy-2,3-difluoro-α-D-talopyranose (14). In a manner similar to Denavit et al. [1], to a stirred solution of 1,6-anhydro-2,3-dideoxy-2,3-difluoro-β-D-mannopyranose **5** (40.1 mg, 0.2414 mmol) in CH_2Cl_2 (1.2 mL, 0.2 M) at 0 °C, were added pyridine (59.0 µL, 0.7242 mmol, 3 equiv) and Tf_2O (61.0 µL, 0.3621 mmol, 1.5 equiv). The mixture was

stirred at room temperature for 30 min and then quenched with water (3 mL). The mixture was extracted with CH_2Cl_2 (3 × 5 mL), and the combined organic phases were successively washed with an aqueous 1 M HCl solution (5 mL) and brine (5 mL). The organic solution was dried over $MgSO_4$, filtered, and concentrated under reduced pressure. The crude triflate **7** was used for the next step without further purification. To a stirred solution of the crude triflate **7** in anhydrous acetonitrile (2.4 mL, 0.1 M) was added tetrabutylammonium bromide (233.5 mg, 0.7242 mmol, 3 equiv). The mixture was stirred at 65 °C for 4 days. The volatiles were removed under reduced pressure and the crude was dissolved in CH_2Cl_2 (2.4 mL, 0.1 M). The mixture cooled to 0 °C and Ac_2O (0.68 mL, 7.242 mmol, 30 equiv) and H_2SO_4 (0.13 mL, 2.414 mmol, 10 equiv) were added. The mixture was stirred at room temperature for 17 h, then cooled to 0 °C. Sodium acetate (396 mg, 4.828 mmol, 20 equiv) was added and the mixture was stirred for an additional 20 min. Water (3 mL) was added and the mixture was extracted with CH_2Cl_2 (3 × 5 mL). The combined organic phases were successively washed with a saturated aqueous $NaHCO_3$ solution (5 mL) and brine (5 mL). The organic solution was dried over $MgSO_4$, filtered, and concentrated under reduced pressure. The crude residue was purified by flash column chromatography (silica gel, EtOAc/Hexanes, 3:7 → 1:1) to give **14** (α/β, >20:1) as a white solid (54.1 mg, 0.1634 mmol, 68% over 3 steps). The resulting product was recrystallized from acetone/heptane to give colorless crystals. R_f = 0.38 (silica, EtOAc/hexanes 1:1); m.p. = 164 – 176 °C; $[\alpha]_D^{25}$ = 73.2 (*c* 0.4, $CHCl_3$); IR (ATR, diamond crystal) *ν* 3016, 2924, 1759, 1728, 1244, 1213, 972 cm^{-1}; ^{1}H NMR (500 MHz, $CDCl_3$) δ 6.36 (ddd, $^3J_{H1–F2}$ = 7.9 Hz, $^3J_{H1–H2}$ = 5.4 Hz, $^4J_{H1–F3}$ = 2.2 Hz, 1H, H1), 4.93 (dddd, $^2J_{H3–F3}$ = 43.7 Hz, $^3J_{H3–F2}$ = 25.7 Hz, $^3J_{H3–H4}$ = 4.4 Hz, $^3J_{H3–H2}$ = 3.1 Hz, 1H, H3), 4.74 (ddddd, $^2J_{H2–F2}$ = 48.7 Hz, $^3J_{H2–H1}$ = 6.2 Hz, $^3J_{H2–H3}$ = 3.2 Hz, $^3J_{H2–F3}$ = 2.2 Hz, 1.1 Hz, 1H, H2), 4.43 (ddt, $^3J_{H4–F3}$ = 4.7 Hz, $^3J_{H4–H3}$ = 4.4 Hz, $^3J_{H4–H5}$ = $^4J_{H4–H6a}$ = 1.5 Hz, 1H, H4), 4.39 (ddd, $^2J_{H6a–H6b}$ = 11.6 Hz, $^3J_{H6a–H5}$ = 6.9 Hz, $^4J_{H6a–H4}$ = 1.4 Hz, 1H, H6a), 4.31 (dd, $^2J_{H6b–H6a}$ = 11.6 Hz, $^3J_{H6b–H5}$ = 5.4 Hz, 1H, H5), 4.18 (dddd, $^3J_{H5–H6a}$ = 6.8 Hz, $^3J_{H5–H6b}$ = 5.4 Hz, 1.6 Hz, $^3J_{H5–H4}$ = 1.5 Hz, 1H, H5), 2.14 (s, 3H, COC*H*$_3$), 2.09 (s, 3H, COC*H*$_3$) ppm; ^{13}C {^{1}H} NMR (126 MHz, $CDCl_3$) δ 170.65 (1C, *C*OCH$_3$), 167.80 (1C, *C*OCH$_3$), 91.12 (dd, $^1J_{C1–F2}$ = 32.4 Hz, $^3J_{C1–F3}$ = 6.7 Hz, 1C, C1), 84.26 (dd, $^1J_{C2–F2}$ = 191.3 Hz, $^2J_{C2–F3}$ = 16.9 Hz, 1C, C2), 83.65 (dd, $^1J_{C3–F3}$ = 196.9 Hz, $^2J_{C3–F2}$ = 15.9 Hz, 1C, C3), 68.96 (d, $^3J_{C5–F3}$ = 4.0 Hz, 1C, C5), 64.60 (d, $^4J_{C6–F3}$ = 4.0 Hz, C6), 44.72 (d, $^2J_{C4–F3}$ = 18.6 Hz, 1C, C4), 20.89 (1C, CO*C*H$_3$), 20.86 (1C, CO*C*H$_3$) ppm; ^{19}F NMR (470 MHz, $CDCl_3$) δ -192.80 (dddt, $^2J_{F3–H3}$ = 44.1 Hz, $^3J_{F3–F2}$ = 16.0 Hz, $^3J_{F3–H4}$ = 3.6 Hz, $^3J_{F3–H1}$ = $^4J_{F3–H1}$ = 2.2 Hz, 1F, F3), -201.62 (dddd, *J* = $^2J_{F2–H2}$ = 49.3 Hz, $^3J_{F2–H3}$ = 25.0 Hz, $^3J_{F2–F3}$ = 15.9 Hz, $^3J_{F2–H1}$ = 8.4 Hz, 1F, F2) ppm; HRMS calcd for $C_{10}H_{17}BrF_2NO_5^+$ $[M + NH_4]^+$ 348.0253 found 348.0256.

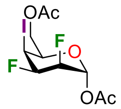

15

1,6-Di-*O*-acetyl-2,3,4-trideoxy-2,3-difluoro-4-iodo-α-D-talopyranose (15). In a manner similar to Denavit et al. [1], to a stirred solution of 1,6-anhydro-2,3-dideoxy-2,3-difluoro-β-D-mannopyranose **5** (44.3 mg, 0.2667 mmol) in CH_2Cl_2 (1.3 mL, 0.2 M) at 0 °C, were added pyridine (64.8 μL, 0.8000 mmol, 3 equiv) and Tf_2O (67.3 μL, 0.4000 mmol, 1.5 equiv). The mixture was stirred at room temperature for 30 min and then quenched with water (3 mL). The mixture was extracted with CH_2Cl_2 (3 × 5 mL), and the combined organic phases were successively washed with an aqueous 1 M HCl solution (5 mL) and brine (5 mL). The organic solution was dried over $MgSO_4$, filtered, and concentrated under reduced pressure. The crude triflate **7** was used for the next step without further purification. To a stirred solution of the crude triflate **7** in anhydrous acetonitrile (2.7 mL, 0.1 M) was added tetrabutylammonium iodide (295.5 mg, 0.8000 mmol, 3 equiv). The mixture was stirred at 65 °C for 11 days. The volatiles were removed under reduced pressure and the crude was dissolved in CH_2Cl_2 (2.7 mL, 0.1 M). The mixture cooled to 0 °C and Ac_2O (0.76 mL, 8.000 mmol, 30 equiv) and H_2SO_4 (0.14 mL, 2.667 mmol, 10 equiv) were added. The mixture was stirred at room temperature for 17 h, then cooled to 0 °C. Sodium acetate (437 mg, 5.334 mmol, 20 equiv) was added and the mixture was stirred for an additional 20 min. Water (3 mL) was added and the mixture was extracted with CH_2Cl_2 (3 × 5 mL). The combined organic phases were successively washed with a saturated aqueous $NaHCO_3$ solution (5 mL) and brine (5 mL). The organic solution was dried over $MgSO_4$, filtered, and concentrated under reduced pressure. The crude residue was purified by flash column chromatography (silica gel, EtOAc/Hexanes, 3:7 → 1:1) to give **15** (α/β, >20:1) as a white solid (49.5 mg, 0.1309 mmol, 49% over 3 steps). The resulting product was recrystallized from acetone/heptane to give colorless crystals. R_f = 0.40 (silica, EtOAc/hexanes 1:1); m.p. = 159.0 – 168.5 °C; $[\alpha]_D^{25}$ = 87.6 (c 0.5, $CHCl_3$); IR (ATR, diamond crystal) ν 2993, 2922, 1761, 1722, 1244, 1018, 970 cm^{-1}; ^1H NMR (500 MHz, $CDCl_3$) δ 6.36 (ddd, $^3J_{H1-F2}$ = 8.0 Hz, $^3J_{H1-H2}$ = 5.0 Hz, $^4J_{H1-F3}$ = 2.2 Hz, 1H, 1H), 4.78 (ddt, $^2J_{H2-F2}$ = 48.5 Hz, $^3J_{H2-H1}$ = 5.7 Hz, $^3J_{H2-H3}$ = $^3J_{H2-F3}$ = 2.7 Hz, 1H, H2), 4.67 (dddd, $^2J_{H3-F3}$ = 44.4 Hz, $^3J_{H3-F2}$ = 24.9 Hz, $^3J_{H3-H4}$ = 3.9 Hz, $^3J_{H3-H2}$ = 2.9 Hz, 1H, H3), 4.47 (dt, $^3J_{H4-F3}$ = 4.0 Hz, $^3J_{H4-H3}$ = $^3J_{H4-H5}$ = 4.0 Hz, 1H, H5), 4.38 (dd, $^2J_{H6a-H6b}$ = 11.8 Hz, $^3J_{H6a-H5}$ = 7.0 Hz, 1H, H6a), 4.24 (dd, $^3J_{H6b-H6a}$ = 11.7 Hz, $^3J_{H6b-H5}$ = 5.1 Hz, 1H, H6b), 3.65 (dd, $^3J_{H5-H6a}$ = 6.3 Hz, $^3J_{H5-H6b}$ = 5.3 Hz, 1H, H5), 2.15 (s, 3H, $COCH_3$), 2.10 (s, 3H, $COCH_3$) ppm; $^{13}C\ \{^1H\}$ NMR (126 MHz, $CDCl_3$) δ 170.65 (1C, $COCH_3$), 167.84 (1C, $COCH_3$), 91.04 (dd, $^2J_{C1-F2}$ = 32.2 Hz, $^3J_{C1-F3}$ = 6.4 Hz, 1C, C1), 84.17 (dd, $^1J_{C3-F3}$ = 191.3 Hz, $^2J_{C3-F2}$ = 16.8 Hz, 1C, C3), 83.83 (dd, $^1J_{C2-F2}$ = 196.0 Hz, $^2J_{C2-F3}$ = 16.3 Hz, 1C, C2), 68.95 (d, $^3J_{C5-F3}$ = 4.4 Hz, 1C, C5), 67.46 (d, $^4J_{C6-F3}$ = 3.7 Hz, 1C, C6), 21.36 (d, $^2J_{C4-F3}$ = 19.0 Hz, 1C, C4), 20.91 (1C, $COCH_3$), 20.87 (1C, $COCH_3$) ppm; ^{19}F NMR (470 MHz, $CDCl_3$) δ -184.56 (dddd, $^2J_{F3-H3}$ = 44.4 Hz, $^3J_{F3-F2}$ = 17.7 Hz, $^3J_{F3-H4}$ = 4.2 Hz, $^3J_{F3-H2}$ = 2.7 Hz, $^4J_{F3-H1}$ = 2.2 Hz, 1F, F3), -200.55 (dddd, $^2J_{F2-H2}$ = 48.6 Hz, $^3J_{F2-H3}$ = 24.9 Hz, $^3J_{F2-F3}$ = 17.7 Hz, $^3J_{F2-H1}$ = 8.0 Hz, 1F, F2) ppm; HRMS calcd for $C_{10}H_{17}F_2INO_5^+$ $[M + NH_4]^+$ 396.0114 found 396.0106.

II. COMPARISON OF ^{19}F RESONANCES OF HALOGENATED TALOSE AND ALLOSE ANALOGUES

Talopyranose analogues **12–15** incorporate a 2,3-*cis*, 3,4-*cis* relationship for the halogens. We previously prepared a small set of trihalogenated allopyranose analogues that also included the 2,3-*cis*, 3,4-*cis* relationship for the halogens (**Figure 1a**) [2]. In order to compare the signals we performed ^{19}F NMR analysis of halogenated allopyranose analogues **4a–d** (**Figure 2**). Unlike analogues **12–15**, compounds **4a–d** adopted a β configuration as the major anomer in acetone-d_6. Moreover, both F2 and F4 are vicinal to the halogen at C3 and undergo an increase in chemical shift depending on the C3 halogen. In all cases, the ^{19}F resonance of F2 occurs at lower field than F4, except for analogue **4d**, whereas the chemical shift is similar. Finally, the chemical shifts of the fluorine atoms next to the chlorine, bromine or iodine atoms appear systematically at lower field for the allose analogues than the talose analogues.

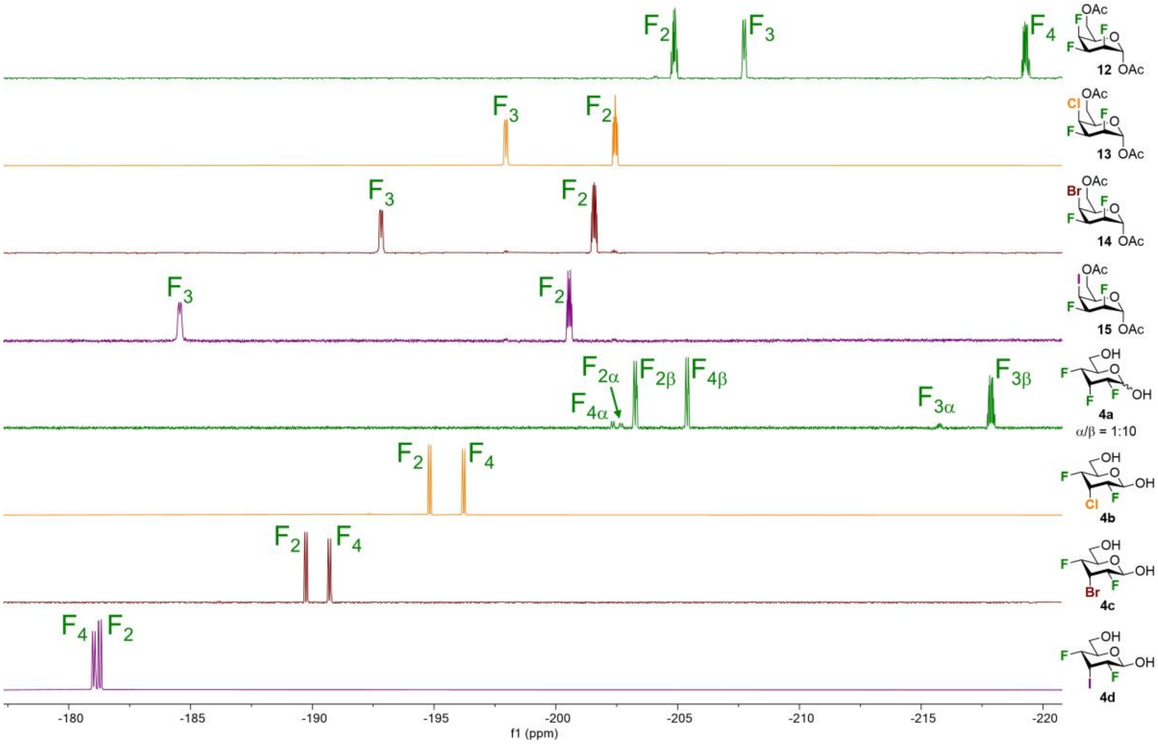

Figure S1. Direct comparison of ^{19}F resonances of halogenated talose analogues **12–15** (^{19}F NMR; 470 MHz, $CDCl_3$) and halogenated allose analogues **4a–d** (^{19}F NMR; 470 MHz, acetone-d_6).

III. Crystal structure determination

Table S1. Crystal data and structure refinement for compound **13**

Empirical formula	$C_{10}H_{13}ClF_2O_5$
Formula weight	286.65
Temperature [K]	150
Crystal system	orthorhombic
Space group (number)	$P2_12_12_1$ (19)
a [Å]	8.7542(4)
b [Å]	9.4335(4)
c [Å]	14.7462(6)
α [°]	90
β [°]	90
γ [°]	90
Volume [Å^3]	1217.78(9)
Z	4
ρ_{calc} [gcm^{-3}]	1.563
μ [mm^{-1}]	2.084
F(000)	592
Crystal size [mm^3]	0.1×0.13×0.25
Crystal colour	clear light colourless
Crystal shape	block
Radiation	Ga K_α (λ=1.34139 Å)
2θ range [°]	9.68 to 137.32 (0.72 Å)
Index ranges	$-12 \le h \le 10$<br>$-13 \le k \le 12$<br>$-20 \le l \le 20$
Reflections collected	48497
Independent reflections	3400<br>R_{int} = 0.0445<br>R_{sigma} = 0.0209
Completeness to θ = 53.594°	99.9 %
Data / Restraints / Parameters	3400 / 0 / 167
Goodness-of-fit on F^2	1.067
Final R indexes [$I \geq 2\sigma(I)$]	R_1 = 0.0259<br>wR_2 = 0.0743
Final R indexes [all data]	R_1 = 0.0261<br>wR_2 = 0.0745
Largest peak/hole [eÅ^{-3}]	0.26/−0.21
Extinction coefficient	0.0042(10)
Flack X parameter	0.170(17)

Table S2. Crystal data and structure refinement for compound **14**

Empirical formula	$C_{10}H_{13}BrF_2O_5$
Formula weight	331.11
Temperature [K]	150
Crystal system	orthorhombic
Space group (number)	$P2_12_12_1$ (19)
a [Å]	8.8122(3)
b [Å]	9.4110(4)
c [Å]	14.8090(6)
α [°]	90
β [°]	90
γ [°]	90
Volume [Å^3]	1228.13(8)
Z	4
ρ_{calc} [gcm^{-3}]	1.791
μ [mm^{-1}]	3.264
F(000)	664
Crystal size [mm^3]	0.16×0.21×0.24
Crystal colour	clear light colourless
Crystal shape	block
Radiation	Ga K_α (λ=1.34139 Å)
2θ range [°]	9.69 to 137.36 (0.72 Å)
Index ranges	$-12 \le h \le 12$<br>$-13 \le k \le 13$<br>$-20 \le l \le 20$
Reflections collected	33417
Independent reflections	3423<br>R_{int} = 0.0375<br>R_{sigma} = 0.0216
Completeness to θ = 53.594°	99.8 %
Data / Restraints / Parameters	3423 / 0 / 166
Goodness-of-fit on F^2	1.130
Final *R* indexes [$I \ge 2\sigma(I)$]	R_1 = 0.0229<br>wR_2 = 0.0621
Final *R* indexes [all data]	R_1 = 0.0229<br>wR_2 = 0.0622
Largest peak/hole [eÅ^{-3}]	0.38/−0.94
Flack X parameter	0.10(2)

Table S3. Crystal data and structure refinement for compound **15**

Empirical formula	$C_{10}H_{13}F_2IO_5$
Formula weight	378.10
Temperature [K]	150
Crystal system	orthorhombic
Space group (number)	$P2_12_12_1$ (19)
a [Å]	8.8808(15)
b [Å]	9.4551(16)
c [Å]	14.927(2)
α [°]	90
β [°]	90
γ [°]	90
Volume [Å^3]	1253.4(4)
Z	4
ρ_{calc} [gcm^{-3}]	2.004
μ [mm^{-1}]	14.021
F(000)	736
Crystal size [mm^3]	0.1×0.14×0.18
Crystal color	clear light colorless
Crystal shape	block
Radiation	Ga K_α (λ=1.34139 Å)
2θ range [°]	9.63 to 121.06 (0.77 Å)
Index ranges	$-11 \leq h \leq 11$<br>$-12 \leq k \leq 12$<br>$-19 \leq l \leq 18$
Reflections collected	22909
Independent reflections	2858<br>R_{int} = 0.0471<br>R_{sigma} = 0.0263
Completeness to θ = 53.594°	99.9 %
Data / Restraints / Parameters	2858 / 0 / 166
Goodness-of-fit on F^2	1.108
Final R indexes [$I \geq 2\sigma(I)$]	R_1 = 0.0651<br>wR_2 = 0.1998
Final R indexes [all data]	R_1 = 0.0660<br>wR_2 = 0.2007
Largest peak/hole [eÅ^{-3}]	1.44/−1.37
Flack X parameter	0.30(3)

Table S4. Crystal data and structure refinement for compound **17**

Empirical formula	$C_{20}H_{15}Br_2F_3O_5$
Formula weight	552.14
Temperature [K]	150
Crystal system	orthorhombic
Space group (number)	$P2_12_12_1$
a [Å]	5.95820(10)
b [Å]	11.7697(3)
c [Å]	28.7068(7)
α [°]	90
β [°]	90
γ [°]	90
Volume [Å^3]	2013.10(8)
Z	4
ρ_{calc} [gcm^{-3}]	1.822
μ [mm^{-1}]	3.726
F(000)	1088.0
Crystal size [mm^3]	0.25×0.16×0.09
Radiation	Ga K_α (λ=1.34139 Å)
2θ range [°]	5.356 to 121.326
Index ranges	$-7 \leq h \leq 7$<br>$-15 \leq k \leq 15$<br>$-37 \leq l \leq 37$
Reflections collected	29233
Independent reflections	4629<br>R_{int} = 0.0320<br>R_{sigma} = 0.0182
Data / Restraints / Parameters	4629 / 0 / 272
Goodness-of-fit on F^2	1.180
Final *R* indexes [$I \geq 2\sigma(I)$]	R_1 = 0.0272<br>wR_2 = 0.0630
Final *R* indexes [all data]	R_1 = 0.0273<br>wR_2 = 0.0632
Largest peak/hole [eÅ^{-3}]	0.47/−0.67
Flack X parameter	-0.032(5)

IV. Crystal packing

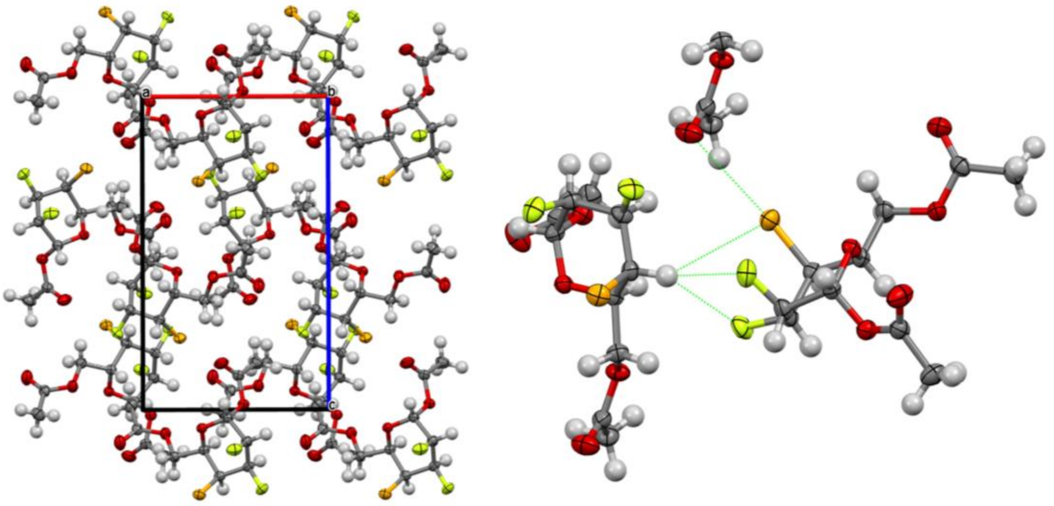

Figure S2. Packing arrangement of compound compound **13**. ORTEP diagram showing 50% thermal ellipsoid probability: carbon (gray), oxygen (red), fluorine (green), chlorine (orange), and hydrogen (white).

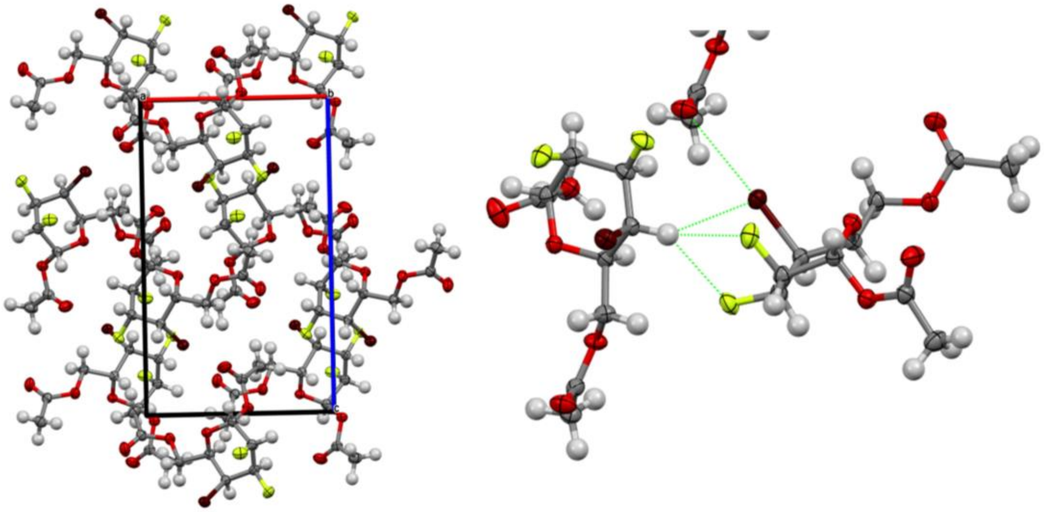

Figure S3. Packing arrangement of compound compound **14**. ORTEP diagram showing 50% thermal ellipsoid probability: carbon (gray), oxygen (red), fluorine (green), bromine (dark red) and hydrogen (white).

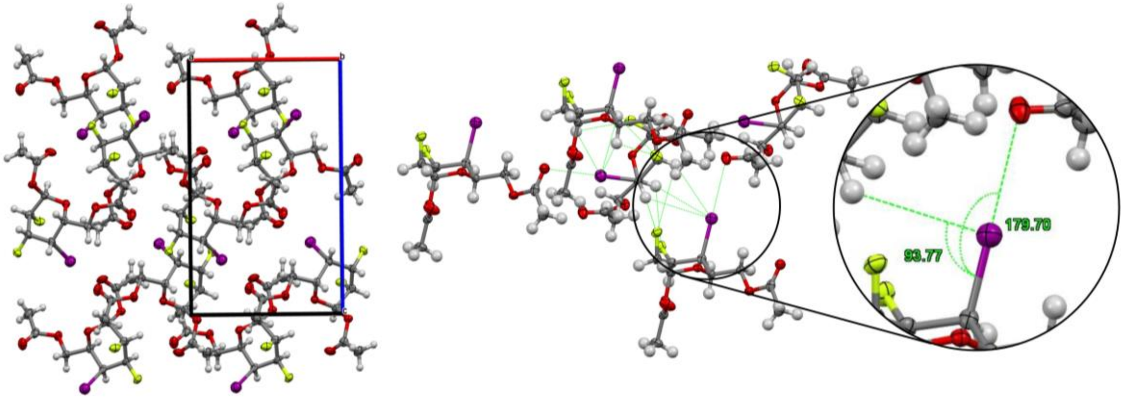

Figure S4. Packing arrangement of compound compound **15**. ORTEP diagram showing 50% thermal ellipsoid probability: carbon (gray), oxygen (red), fluorine (green), iodine (purple), and hydrogen (white).

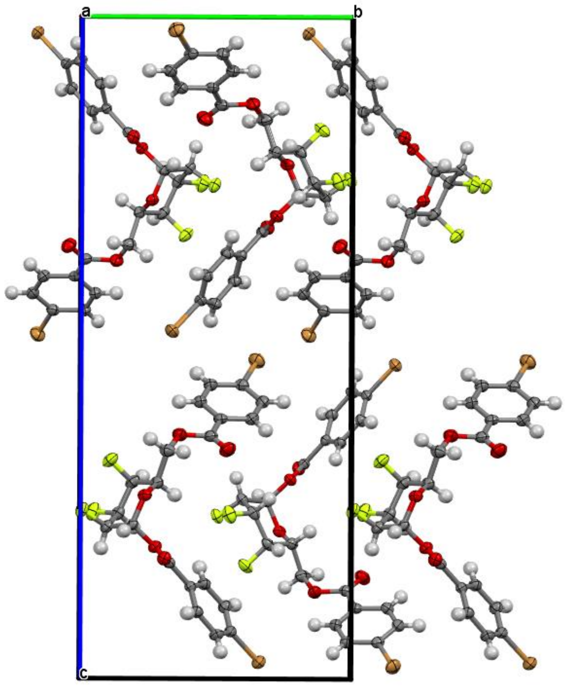

Figure S5. Packing arrangement of compound compound **17**. ORTEP diagram showing 50% thermal ellipsoid probability: carbon (gray), oxygen (red), fluorine (green), and hydrogen (white).

V. Density functional theory calculations

DFT computations were performed using Gaussian 16 revision B.01 [3] with the CAM-B3LYP [4,5,6] functional and the Def2TZVP basis set [7], which includes effective core potentials for iodine. Empirical dispersion was accounted with Grimme's D3 [8,9] correction including Becke-Johnson damping [10]. Computations were performed both in the gas phase (i.e. individual molecules with thermal corrections based on ideal gas assumptions) and in a chloroform solution, using the polarizable continuum model (PCM) [11].

Table S5. Dipoles (Debye) of $^{4}C_{1}$ and $^{1}C_{4}$ structures in gas phase and chloroform (PCM) computed from CAM-B3LYP-D3BJ/Def2TZVP.

Entry	X	Gas phase $^{1}C_{4}$	$CHCl_3$ (PCM) $^{1}C_{4}$	Gas phase $^{4}C_{1}$	$CHCl_3$ (PCM) $^{4}C_{1}$
1	F	4.55	5.95	6.48	8.09
2	Cl	4.45	5.93	6.34	7.93
3	Br	4.40	5.89	6.30	7.94
4	I	4.32	5.78	6.07	7.64

Table S6. Optimized structure of $^{4}C_{1}$ **X=F** CAM-B3LYP-D3BJ/Def2TZVP

6	-0.444987000	-0.161669000	-0.013556000
6	-0.509661000	1.288132000	0.444886000
6	0.867504000	1.725589000	0.888767000
6	1.922046000	1.470049000	-0.164654000
6	1.843272000	0.022986000	-0.636910000
8	0.560729000	-0.354555000	-0.995416000
6	-1.729482000	-0.628822000	-0.656171000
8	-2.738200000	-0.544998000	0.349494000
9	-0.959716000	2.088614000	-0.583789000
9	0.864265000	3.053149000	1.249848000
9	1.726125000	2.280931000	-1.257485000
8	2.325040000	-0.771166000	0.445236000
6	-3.973093000	-0.934978000	-0.021457000
6	-4.955102000	-0.756809000	1.094162000
8	-4.218457000	-1.359796000	-1.113217000
6	2.979104000	-1.923314000	0.126846000
6	3.331586000	-2.686712000	1.362995000
8	3.224923000	-2.245225000	-0.994852000
1	-0.236559000	-0.781461000	0.865138000
1	-1.219060000	1.383864000	1.268156000
1	1.134582000	1.136369000	1.770817000
1	2.915882000	1.677687000	0.234138000
1	2.463932000	-0.129114000	-1.515196000
1	-1.988144000	0.001188000	-1.504535000
1	-1.622993000	-1.654947000	-1.003070000
1	-4.596787000	-1.254630000	1.994048000
1	-5.056818000	0.304184000	1.322148000
1	-5.916899000	-1.161929000	0.796822000
1	3.894067000	-2.051946000	2.046341000
1	2.418853000	-2.992633000	1.874219000
1	3.914471000	-3.561664000	1.094403000

Table S7. Optimized structure of $^{4}\boldsymbol{C_1}$ **X=Cl** CAM-B3LYP-D3BJ/Def2TZVP

6	-0.345676000	-0.309832000	0.014589000
6	-0.518093000	1.111206000	0.546635000
6	0.839756000	1.589091000	1.025133000
6	1.935485000	1.436200000	-0.008319000
6	1.922198000	0.026687000	-0.588515000
8	0.657978000	-0.381150000	-0.981842000
6	-1.585546000	-0.901726000	-0.609339000
8	-2.598158000	-0.863953000	0.393984000
17	-1.249434000	2.211093000	-0.650356000
9	0.788138000	2.886504000	1.474338000
9	1.765110000	2.321051000	-1.046732000
8	2.436810000	-0.825450000	0.431801000
6	-3.815868000	-1.300636000	0.018039000
6	-4.810648000	-1.148706000	1.125903000
8	-4.038512000	-1.740518000	-1.072562000
6	3.139299000	-1.921412000	0.028569000
6	3.524987000	-2.760435000	1.204206000
8	3.397224000	-2.146423000	-1.113771000
1	-0.057919000	-0.929310000	0.871838000
1	-1.207160000	1.089348000	1.386534000
1	1.116158000	0.956826000	1.875065000
1	2.906055000	1.638896000	0.446259000
1	2.551112000	-0.028383000	-1.472322000
1	-1.894303000	-0.329276000	-1.480733000
1	-1.393644000	-1.928238000	-0.917269000
1	-4.434852000	-1.611953000	2.036950000
1	-4.961591000	-0.089012000	1.332147000
1	-5.751551000	-1.602267000	0.831722000
1	4.057483000	-2.155274000	1.936635000
1	2.626449000	-3.145313000	1.686483000
1	4.146808000	-3.585297000	0.872008000

Table S8. Optimized structure of $^{4}C_{1}$ **X=Br** CAM-B3LYP-D3BJ/Def2TZVP

6	0.111587000	0.597503000	0.064537000
6	0.451899000	-0.746764000	0.700769000
6	-0.844006000	-1.364204000	1.188186000
6	-1.928755000	-1.421448000	0.133055000
6	-2.078636000	-0.066425000	-0.548768000
8	-0.866198000	0.469988000	-0.951438000
6	1.274273000	1.318394000	-0.572250000
8	2.263727000	1.462501000	0.443777000
35	1.459045000	-1.924139000	-0.479034000
9	-0.643513000	-2.610445000	1.730608000
9	-1.629215000	-2.349382000	-0.836398000
8	-2.721337000	0.783653000	0.397530000
6	3.430200000	2.013608000	0.055219000
6	4.416614000	2.040548000	1.180324000
8	3.619773000	2.412037000	-1.057276000
6	-3.546381000	1.749435000	-0.096693000
6	-4.063429000	2.614639000	1.007275000
8	-3.802348000	1.856971000	-1.256401000
1	-0.281349000	1.225804000	0.872965000
1	1.113524000	-0.580519000	1.544940000
1	-1.218600000	-0.716235000	1.987888000
1	-2.875660000	-1.711452000	0.590060000
1	-2.687317000	-0.155223000	-1.443928000
1	1.672191000	0.752198000	-1.410953000
1	0.953942000	2.294718000	-0.932545000
1	3.972860000	2.502102000	2.061119000
1	4.689181000	1.018595000	1.444223000
1	5.301777000	2.587509000	0.872232000
1	-4.533122000	2.000751000	1.774562000
1	-3.232648000	3.144685000	1.473068000
1	-4.775941000	3.327443000	0.605202000

Table S9. Optimized structure of $^{4}\boldsymbol{C_1}$ **X=I** CAM-B3LYP-D3BJ/Def2TZVP

6	-0.211679000	0.807140000	0.097086000
6	0.327557000	-0.434644000	0.800600000
6	-0.865638000	-1.228402000	1.295099000
6	-1.902147000	-1.505408000	0.226333000
6	-2.249127000	-0.226374000	-0.526085000
8	-1.127519000	0.478715000	-0.932161000
6	0.824758000	1.696539000	-0.544648000
8	1.765060000	2.021254000	0.475741000
53	1.708491000	-1.591402000	-0.376547000
9	-0.487116000	-2.403823000	1.900185000
9	-1.432029000	-2.416227000	-0.691761000
8	-3.045540000	0.552222000	0.362846000
6	2.848396000	2.717215000	0.078586000
6	3.805766000	2.918242000	1.210906000
8	2.996061000	3.100271000	-1.045554000
6	-3.999395000	1.349099000	-0.196240000
6	-4.678685000	2.170405000	0.851932000
8	-4.236261000	1.360296000	-1.364937000
1	-0.729300000	1.391026000	0.868406000
1	0.926950000	-0.122482000	1.649583000
1	-1.359394000	-0.616888000	2.058396000
1	-2.801347000	-1.924207000	0.679414000
1	-2.811152000	-0.454617000	-1.427075000
1	1.320564000	1.187859000	-1.368316000
1	0.353596000	2.599788000	-0.929477000
1	3.288059000	3.328899000	2.076323000
1	4.223575000	1.954216000	1.501761000
1	4.603959000	3.582813000	0.896508000
1	-5.065646000	1.525368000	1.639585000
1	-3.956499000	2.848193000	1.307062000
1	-5.484317000	2.739828000	0.400113000

Table S10. Optimized structure of $^{1}\boldsymbol{C_4}$ **X=F** CAM-B3LYP-D3BJ/Def2TZVP

6	2.422267000	-0.157048000	-0.920780000
6	2.450880000	0.768016000	0.279919000
6	1.305681000	1.757062000	0.192478000
6	-0.012012000	1.034718000	-0.022146000
8	0.097751000	0.237032000	-1.172471000
6	1.054458000	-0.808834000	-1.119909000
6	0.729335000	-1.893925000	-0.106785000
9	3.406910000	-1.115207000	-0.788096000
9	2.318628000	0.030789000	1.442176000
9	1.263738000	2.507410000	1.343164000
8	-0.557258000	-2.413985000	-0.419470000
8	-1.280724000	-1.961134000	1.658673000
6	-1.502656000	-2.341898000	0.544760000
6	-2.834565000	-2.773260000	0.025685000
8	-0.973333000	2.016033000	-0.299924000
8	-2.624709000	0.532833000	0.043096000
6	-2.276204000	1.640422000	-0.236167000
6	-3.169965000	2.793117000	-0.561033000
1	2.650477000	0.433419000	-1.811319000
1	3.407418000	1.289161000	0.342535000
1	1.461634000	2.432072000	-0.652498000
1	-0.312514000	0.451720000	0.850936000
1	1.017908000	-1.254084000	-2.113572000
1	0.740660000	-1.541663000	0.918377000
1	1.458571000	-2.696642000	-0.205474000
1	-3.453936000	-3.107531000	0.852757000
1	-3.301301000	-1.900443000	-0.431582000
1	-2.733031000	-3.551878000	-0.726267000
1	-4.206319000	2.486526000	-0.464101000
1	-2.957041000	3.625781000	0.108117000
1	-2.973127000	3.130179000	-1.578459000

Table S11. Optimized structure of $^{1}\boldsymbol{C_4}$ **X=Cl** CAM-B3LYP-D3BJ/Def2TZVP

6	-2.223159000	-0.334945000	-0.770739000
6	-2.018888000	-1.254891000	0.418785000
6	-0.706794000	-2.002095000	0.261331000
6	0.438437000	-1.045433000	-0.010349000
8	0.118016000	-0.270863000	-1.136604000
6	-1.014467000	0.575022000	-1.010567000
6	-0.823862000	1.684670000	0.012089000
17	-3.747927000	0.575973000	-0.621593000
9	-1.975667000	-0.525880000	1.591404000
9	-0.463425000	-2.733638000	1.398970000
8	0.339633000	2.416987000	-0.352337000
8	1.222313000	2.096171000	1.688132000
6	1.322745000	2.515794000	0.570499000
6	2.527932000	3.188845000	0.001305000
8	1.546899000	-1.829443000	-0.359563000
8	2.917851000	-0.076092000	-0.059837000
6	2.761399000	-1.223915000	-0.350266000
6	3.830881000	-2.188770000	-0.748078000
1	-2.333378000	-0.960587000	-1.654347000
1	-2.845440000	-1.960166000	0.511818000
1	-0.777300000	-2.692768000	-0.582356000
1	0.675819000	-0.425956000	0.857002000
1	-1.114431000	1.034714000	-1.992872000
1	-0.722382000	1.318237000	1.027516000
1	-1.677109000	2.358525000	-0.028740000
1	3.123075000	3.606768000	0.807764000
1	3.114779000	2.425798000	-0.510280000
1	2.248803000	3.956421000	-0.716445000
1	4.797987000	-1.699487000	-0.695854000
1	3.809235000	-3.056213000	-0.089652000
1	3.643885000	-2.540926000	-1.762230000

Table S12. Optimized structure of ${}^{1}\boldsymbol{C_4}$ **X=Br** CAM-B3LYP-D3BJ/Def2TZVP

6	-1.775625000	-0.555975000	-0.622339000
6	-1.439875000	-1.464699000	0.543586000
6	-0.076359000	-2.094485000	0.314615000
6	0.968208000	-1.041278000	-0.000406000
8	0.527680000	-0.287460000	-1.099837000
6	-0.665270000	0.458801000	-0.906780000
6	-0.511518000	1.569976000	0.120808000
35	-3.520214000	0.272402000	-0.389454000
9	-1.401829000	-0.747992000	1.724189000
9	0.283751000	-2.811032000	1.430464000
8	0.572057000	2.394598000	-0.289190000
8	1.566163000	2.139896000	1.708729000
6	1.582428000	2.572039000	0.591473000
6	2.700289000	3.348268000	-0.022783000
8	2.120668000	-1.724753000	-0.412929000
8	3.352219000	0.134693000	-0.150636000
6	3.279121000	-1.018987000	-0.450762000
6	4.405793000	-1.885500000	-0.911888000
1	-1.881176000	-1.176857000	-1.508342000
1	-2.194072000	-2.242532000	0.666130000
1	-0.129345000	-2.782181000	-0.532726000
1	1.194898000	-0.411363000	0.862250000
1	-0.850699000	0.918251000	-1.876561000
1	-0.332427000	1.202279000	1.125091000
1	-1.414842000	2.176229000	0.131159000
1	3.292116000	3.811120000	0.761284000
1	3.327089000	2.640950000	-0.565982000
1	2.325958000	4.093176000	-0.720805000
1	5.329859000	-1.316916000	-0.896766000
1	4.487925000	-2.758999000	-0.266365000
1	4.201448000	-2.240771000	-1.921594000

Table S13. Optimized structure of $^{1}\boldsymbol{C_4}$ **X=I** CAM-B3LYP-D3BJ/Def2TZVP

6	-1.370677000	-0.644607000	-0.554344000
6	-0.973356000	-1.549255000	0.594868000
6	0.408437000	-2.125923000	0.332655000
6	1.406268000	-1.035379000	-0.002885000
8	0.913159000	-0.294147000	-1.087797000
6	-0.301808000	0.408072000	-0.860507000
6	-0.152161000	1.518545000	0.168832000
53	-3.335280000	0.174233000	-0.274247000
9	-0.936047000	-0.842013000	1.782764000
9	0.821236000	-2.832438000	1.436920000
8	0.896981000	2.375120000	-0.263670000
8	1.930182000	2.168638000	1.719743000
6	1.913818000	2.594012000	0.599891000
6	2.993184000	3.406115000	-0.036492000
8	2.572782000	-1.675561000	-0.445568000
8	3.746050000	0.222179000	-0.190849000
6	3.705150000	-0.930495000	-0.501311000
6	4.850268000	-1.753347000	-0.995903000
1	-1.476335000	-1.262893000	-1.441930000
1	-1.689847000	-2.360001000	0.729145000
1	0.359859000	-2.812629000	-0.515697000
1	1.631394000	-0.401379000	0.857182000
1	-0.524915000	0.866785000	-1.822694000
1	0.061906000	1.150815000	1.166430000
1	-1.070469000	2.101016000	0.206885000
1	3.584256000	3.889562000	0.735604000
1	3.632304000	2.719647000	-0.591849000
1	2.580385000	4.137304000	-0.727356000
1	5.755056000	-1.154368000	-0.992619000
1	4.974740000	-2.631230000	-0.363254000
1	4.636831000	-2.103191000	-2.005638000

Table S14. Optimized structure of $^{4}C_{1}$ **X=F** CAM-B3LYP-D3BJ/Def2TZVP in $CHCl_3$ (PCM)

6	-0.423988000	-0.152866000	-0.016781000
6	-0.485584000	1.293242000	0.448339000
6	0.891114000	1.728572000	0.893389000
6	1.952227000	1.466915000	-0.150897000
6	1.865396000	0.025106000	-0.635892000
8	0.578661000	-0.334008000	-1.007095000
6	-1.709691000	-0.620132000	-0.657609000
8	-2.718313000	-0.535360000	0.347822000
9	-0.930217000	2.101965000	-0.585095000
9	0.885757000	3.066611000	1.234126000
9	1.763120000	2.288164000	-1.245333000
8	2.333823000	-0.776782000	0.441184000
6	-3.956439000	-0.904513000	-0.016976000
6	-4.928617000	-0.738850000	1.106102000
8	-4.213339000	-1.308379000	-1.118765000
6	2.890320000	-1.976537000	0.127352000
6	3.266960000	-2.725115000	1.361896000
8	3.041660000	-2.344241000	-1.000990000
1	-0.208738000	-0.776287000	0.856258000
1	-1.197465000	1.392371000	1.267656000
1	1.151217000	1.153880000	1.785456000
1	2.945158000	1.668161000	0.249886000
1	2.489534000	-0.120655000	-1.512695000
1	-1.970056000	0.008571000	-1.506497000
1	-1.601542000	-1.647439000	-0.999813000
1	-4.577742000	-1.277059000	1.985403000
1	-5.000135000	0.315902000	1.370802000
1	-5.902927000	-1.108869000	0.804192000
1	3.926872000	-2.115542000	1.977655000
1	2.370895000	-2.933856000	1.946071000
1	3.756857000	-3.654611000	1.091558000

Table S15. Optimized structure of $^{4}C_{1}$ **X=Cl** CAM-B3LYP-D3BJ/Def2TZVP in $CHCl_3$ (PCM)

6	-0.328161000	-0.301660000	0.015097000
6	-0.496769000	1.116955000	0.551749000
6	0.862192000	1.596235000	1.024943000
6	1.960345000	1.434893000	-0.003581000
6	1.940302000	0.028325000	-0.588866000
8	0.670953000	-0.364426000	-0.989132000
6	-1.569148000	-0.893839000	-0.607323000
8	-2.584898000	-0.848884000	0.392489000
17	-1.230968000	2.221363000	-0.648310000
9	0.809279000	2.905562000	1.453356000
9	1.789887000	2.323352000	-1.046707000
8	2.445449000	-0.826344000	0.428616000
6	-3.802913000	-1.277843000	0.025055000
6	-4.789097000	-1.139044000	1.139314000
8	-4.033748000	-1.707768000	-1.072554000
6	3.061446000	-1.970761000	0.029876000
6	3.479879000	-2.785656000	1.207609000
8	3.228426000	-2.247879000	-1.121813000
1	-0.034665000	-0.923131000	0.867511000
1	-1.184970000	1.098883000	1.391725000
1	1.134822000	0.979197000	1.885513000
1	2.931540000	1.635681000	0.447620000
1	2.570752000	-0.023849000	-1.471655000
1	-1.875039000	-0.325276000	-1.482435000
1	-1.377022000	-1.922823000	-0.905687000
1	-4.419053000	-1.644207000	2.030254000
1	-4.911734000	-0.084133000	1.383997000
1	-5.742888000	-1.559879000	0.838445000
1	4.112050000	-2.189281000	1.864080000
1	2.598340000	-3.079777000	1.776906000
1	4.013911000	-3.668027000	0.871064000

Table S16. Optimized structure of $^{4}C_{1}$ **X=Br** CAM-B3LYP-D3BJ/Def2TZVP in $CHCl_3$ (PCM)

6	0.092701000	0.589197000	0.061462000
6	0.435905000	-0.750033000	0.704518000
6	-0.857008000	-1.377172000	1.186869000
6	-1.944522000	-1.433419000	0.135406000
6	-2.094979000	-0.081692000	-0.551463000
8	-0.878526000	0.446687000	-0.961295000
6	1.253726000	1.314945000	-0.573738000
8	2.243406000	1.460859000	0.442081000
35	1.453642000	-1.926181000	-0.476946000
9	-0.645507000	-2.634655000	1.710563000
9	-1.638964000	-2.364471000	-0.837371000
8	-2.731446000	0.768111000	0.393493000
6	3.410514000	2.006944000	0.064633000
6	4.379585000	2.060835000	1.200982000
8	3.613929000	2.388672000	-1.055907000
6	-3.479615000	1.792179000	-0.096232000
6	-4.028260000	2.624992000	1.013430000
8	-3.652092000	1.964373000	-1.267337000
1	-0.309313000	1.218342000	0.863075000
1	1.096212000	-0.582899000	1.548914000
1	-1.230201000	-0.745012000	1.998095000
1	-2.890721000	-1.726633000	0.588929000
1	-2.705471000	-0.176333000	-1.444721000
1	1.654063000	0.751385000	-1.413127000
1	0.928499000	2.291233000	-0.928783000
1	3.937972000	2.594187000	2.041610000
1	4.604732000	1.047821000	1.533581000
1	5.291202000	2.555030000	0.881580000
1	-4.606018000	1.999867000	1.692975000
1	-3.206212000	3.057950000	1.582876000
1	-4.652420000	3.413258000	0.605897000

Table S17. Optimized structure of $^{4}C_{1}$ **X=I** CAM-B3LYP-D3BJ/Def2TZVP in $CHCl_3$ (PCM)

6	-0.225726000	0.790986000	0.097001000
6	0.326124000	-0.442357000	0.803424000
6	-0.858147000	-1.245470000	1.303695000
6	-1.902930000	-1.529128000	0.245506000
6	-2.255468000	-0.259919000	-0.520036000
8	-1.132863000	0.442924000	-0.935775000
6	0.799350000	1.694376000	-0.543941000
8	1.736492000	2.030178000	0.476446000
53	1.709711000	-1.590985000	-0.385504000
9	-0.462084000	-2.427400000	1.894970000
9	-1.430071000	-2.448682000	-0.670697000
8	-3.052986000	0.520162000	0.360613000
6	2.806290000	2.745580000	0.093808000
6	3.744297000	2.974981000	1.234253000
8	2.959894000	3.127118000	-1.034695000
6	-3.947282000	1.374346000	-0.204055000
6	-4.662271000	2.161533000	0.842399000
8	-4.109969000	1.451223000	-1.386725000
1	-0.754134000	1.368070000	0.864309000
1	0.928763000	-0.126351000	1.648251000
1	-1.344470000	-0.644563000	2.078356000
1	-2.798513000	-1.948197000	0.702892000
1	-2.815408000	-0.503872000	-1.418089000
1	1.302050000	1.193251000	-1.368211000
1	0.314630000	2.592287000	-0.923367000
1	3.206433000	3.385147000	2.087352000
1	4.174100000	2.021301000	1.540977000
1	4.536004000	3.650459000	0.927218000
1	-5.124717000	1.487436000	1.561957000
1	-3.945469000	2.779451000	1.382865000
1	-5.414492000	2.790088000	0.377499000

Table S18. Optimized structure of $^{1}\boldsymbol{C_4}$ **X=F** CAM-B3LYP-D3BJ/Def2TZVP in $CHCl_3$ (PCM)

6	-2.414879000	0.363537000	-0.898895000
6	-2.518402000	-0.587026000	0.275967000
6	-1.477364000	-1.679996000	0.148070000
6	-0.102855000	-1.071876000	-0.052701000
8	-0.138824000	-0.251584000	-1.191660000
6	-0.992494000	0.880851000	-1.113449000
6	-0.536427000	1.909699000	-0.094553000
9	-3.290136000	1.418038000	-0.710557000
9	-2.288459000	0.107813000	1.456426000
9	-1.492479000	-2.456179000	1.287761000
8	0.783727000	2.305123000	-0.460944000
8	1.489528000	2.113356000	1.659870000
6	1.726404000	2.310866000	0.498281000
6	3.079981000	2.567377000	-0.076900000
8	0.788700000	-2.114429000	-0.331765000
8	2.519843000	-0.784715000	0.189631000
6	2.111340000	-1.848976000	-0.176483000
6	2.934917000	-3.043471000	-0.521944000
1	-2.724815000	-0.170306000	-1.798692000
1	-3.520649000	-1.009335000	0.346049000
1	-1.707419000	-2.318866000	-0.706399000
1	0.232031000	-0.526278000	0.830609000
1	-0.931537000	1.334078000	-2.101604000
1	-0.536104000	1.538797000	0.923897000
1	-1.189908000	2.779128000	-0.144296000
1	3.751981000	2.909724000	0.704087000
1	3.451063000	1.623048000	-0.476586000
1	3.030509000	3.289390000	-0.888421000
1	3.984064000	-2.825892000	-0.351587000
1	2.622349000	-3.894010000	0.082342000
1	2.772975000	-3.304455000	-1.567474000

Table S19. Optimized structure of $^{1}C_{4}$ **X=Cl** CAM-B3LYP-D3BJ/Def2TZVP in $CHCl_3$ (PCM)

6	-2.240092000	-0.291458000	-0.765819000
6	-2.054962000	-1.243789000	0.399725000
6	-0.775839000	-2.038172000	0.210653000
6	0.394137000	-1.109288000	-0.041494000
8	0.101360000	-0.313582000	-1.160381000
6	-1.000244000	0.572768000	-1.016506000
6	-0.739128000	1.664764000	0.007132000
17	-3.718063000	0.686361000	-0.559176000
9	-1.956175000	-0.533818000	1.587914000
9	-0.544915000	-2.796107000	1.339234000
8	0.439377000	2.348765000	-0.412617000
8	1.223005000	2.400413000	1.687837000
6	1.374958000	2.615188000	0.514971000
6	2.608334000	3.179951000	-0.108619000
8	1.499637000	-1.897288000	-0.383549000
8	2.878677000	-0.201236000	0.125244000
6	2.723930000	-1.321742000	-0.267538000
6	3.795648000	-2.267841000	-0.692427000
1	-2.401252000	-0.891621000	-1.658596000
1	-2.908367000	-1.913671000	0.497343000
1	-0.880224000	-2.712936000	-0.640517000
1	0.628686000	-0.506673000	0.837019000
1	-1.098828000	1.042014000	-1.993846000
1	-0.604682000	1.287822000	1.014743000
1	-1.568851000	2.368366000	0.012511000
1	3.197812000	3.693922000	0.644410000
1	3.187319000	2.345955000	-0.506451000
1	2.360637000	3.849550000	-0.928802000
1	4.768087000	-1.811483000	-0.540231000
1	3.718484000	-3.193274000	-0.123532000
1	3.661168000	-2.515639000	-1.745132000

Table S20. Optimized structure of [1]C_4 **X=Br** CAM-B3LYP-D3BJ/Def2TZVP in $CHCl_3$ (PCM)

6	-1.783743000	-0.566329000	-0.633019000
6	-1.451764000	-1.501210000	0.511714000
6	-0.103576000	-2.153339000	0.258828000
6	0.948798000	-1.103645000	-0.032980000
8	0.524165000	-0.335470000	-1.128434000
6	-0.657916000	0.429230000	-0.924834000
6	-0.460249000	1.533878000	0.099784000
35	-3.502416000	0.302524000	-0.350005000
9	-1.376305000	-0.796635000	1.704948000
9	0.255851000	-2.889211000	1.368047000
8	0.627146000	2.331537000	-0.362490000
8	1.463690000	2.487044000	1.711825000
6	1.557092000	2.704635000	0.533322000
6	2.705055000	3.388737000	-0.132433000
8	2.115624000	-1.767163000	-0.431334000
8	3.329433000	0.059562000	0.044651000
6	3.276867000	-1.067832000	-0.355523000
6	4.423947000	-1.893500000	-0.831569000
1	-1.923955000	-1.169761000	-1.525612000
1	-2.220800000	-2.262180000	0.637076000
1	-0.173815000	-2.830115000	-0.594368000
1	1.156912000	-0.486266000	0.841922000
1	-0.847226000	0.893599000	-1.890943000
1	-0.246445000	1.164319000	1.096427000
1	-1.352739000	2.153892000	0.149754000
1	3.259279000	3.970268000	0.597910000
1	3.356301000	2.615548000	-0.541297000
1	2.363832000	4.019388000	-0.949905000
1	5.347718000	-1.335641000	-0.719703000
1	4.471282000	-2.821395000	-0.263288000
1	4.270751000	-2.155228000	-1.878260000

Table S21. Optimized structure of ^{1}***C*****4 X=I** CAM-B3LYP-D3BJ/Def2TZVP in $CHCl_3$ (PCM)

6	-1.376478000	-0.666741000	-0.575036000
6	-0.978547000	-1.596905000	0.551941000
6	0.391373000	-2.188989000	0.266546000
6	1.392035000	-1.095074000	-0.040402000
8	0.913331000	-0.340731000	-1.122406000
6	-0.296538000	0.372803000	-0.887257000
6	-0.111813000	1.479311000	0.138171000
53	-3.318580000	0.186774000	-0.242151000
9	-0.906935000	-0.902476000	1.753018000
9	0.805946000	-2.916296000	1.362622000
8	0.937959000	2.314833000	-0.343070000
8	1.790517000	2.520848000	1.720027000
6	1.860837000	2.734200000	0.539098000
6	2.969517000	3.464021000	-0.144564000
8	2.578699000	-1.706474000	-0.464274000
8	3.725910000	0.161912000	0.015180000
6	3.712264000	-0.963086000	-0.395137000
6	4.885681000	-1.739462000	-0.889727000
1	-1.512739000	-1.269338000	-1.468719000
1	-1.706864000	-2.395166000	0.687599000
1	0.330041000	-2.863060000	-0.589467000
1	1.591485000	-0.473943000	0.833842000
1	-0.524143000	0.834716000	-1.846290000
1	0.134932000	1.111802000	1.128114000
1	-1.021842000	2.070943000	0.212484000
1	3.508368000	4.071324000	0.576249000
1	3.647395000	2.718164000	-0.560715000
1	2.590216000	4.076822000	-0.958851000
1	5.786369000	-1.142296000	-0.793591000
1	4.982189000	-2.662690000	-0.319873000
1	4.726922000	-2.010925000	-1.933006000

VI. NMR spectra of compounds

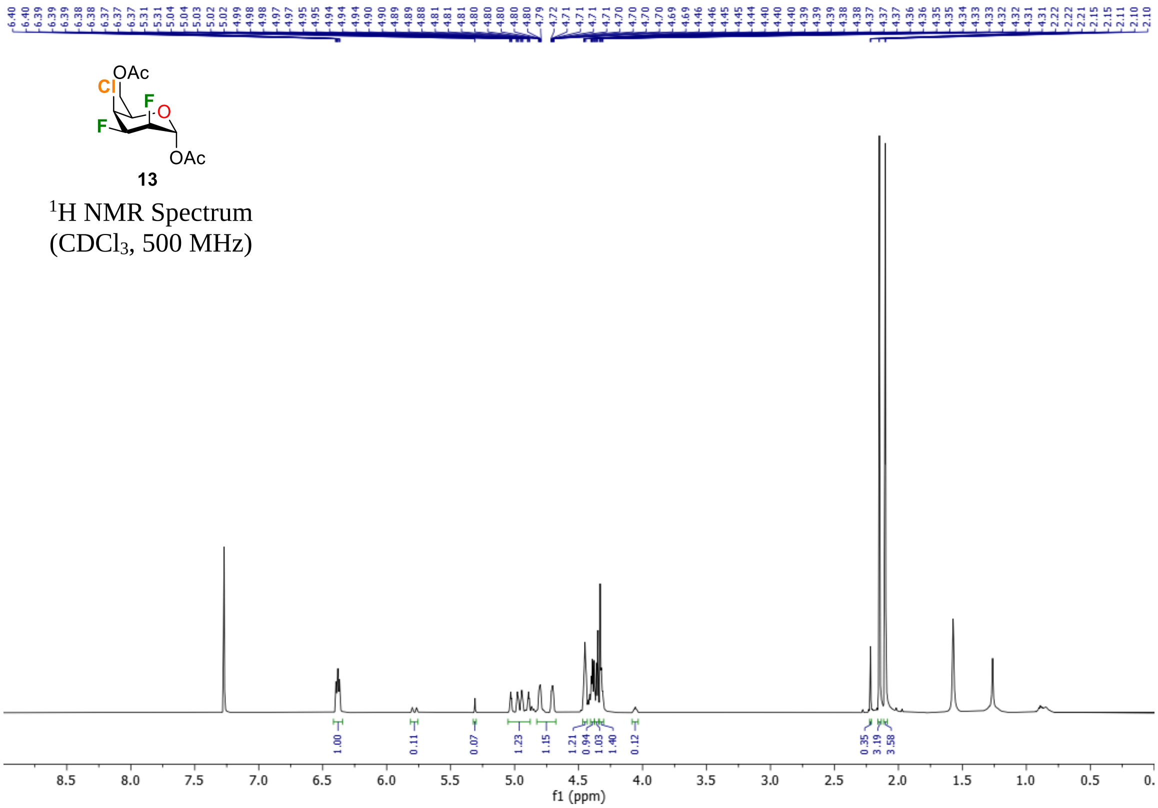

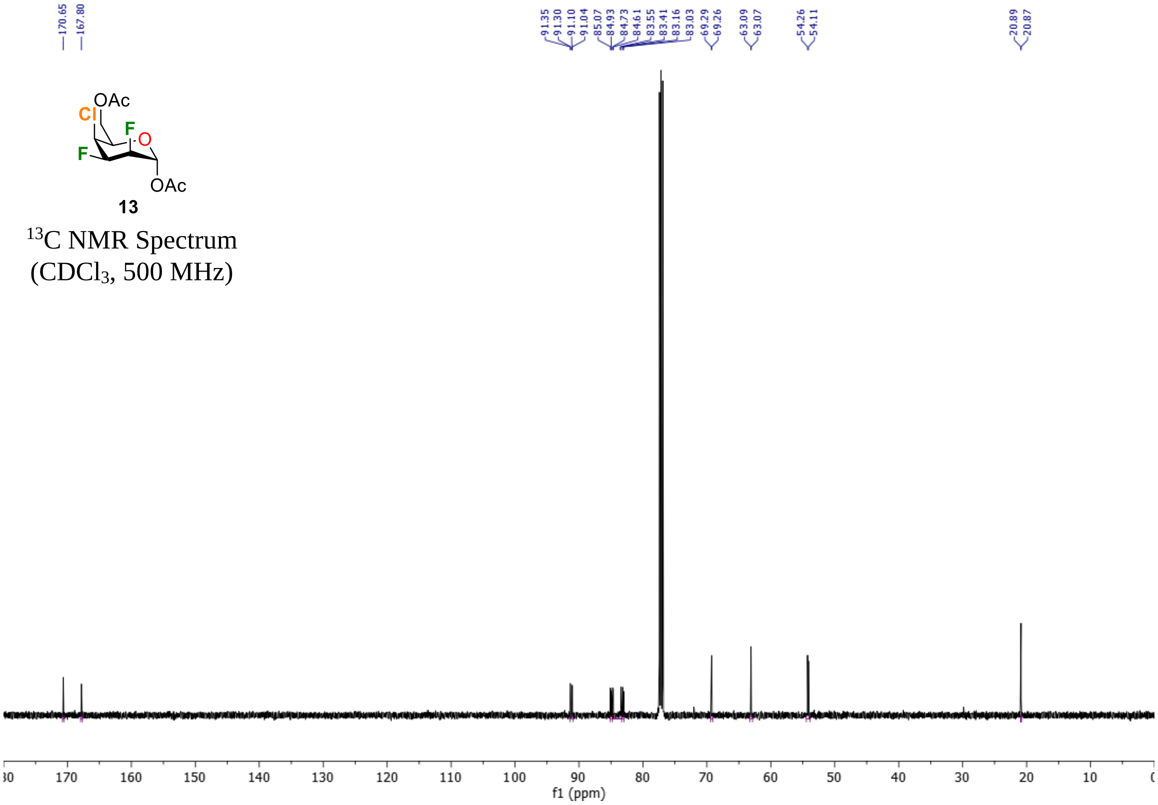

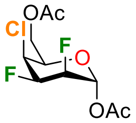

13

^{19}F NMR Spectrum
($CDCl_3$, 500 MHz)

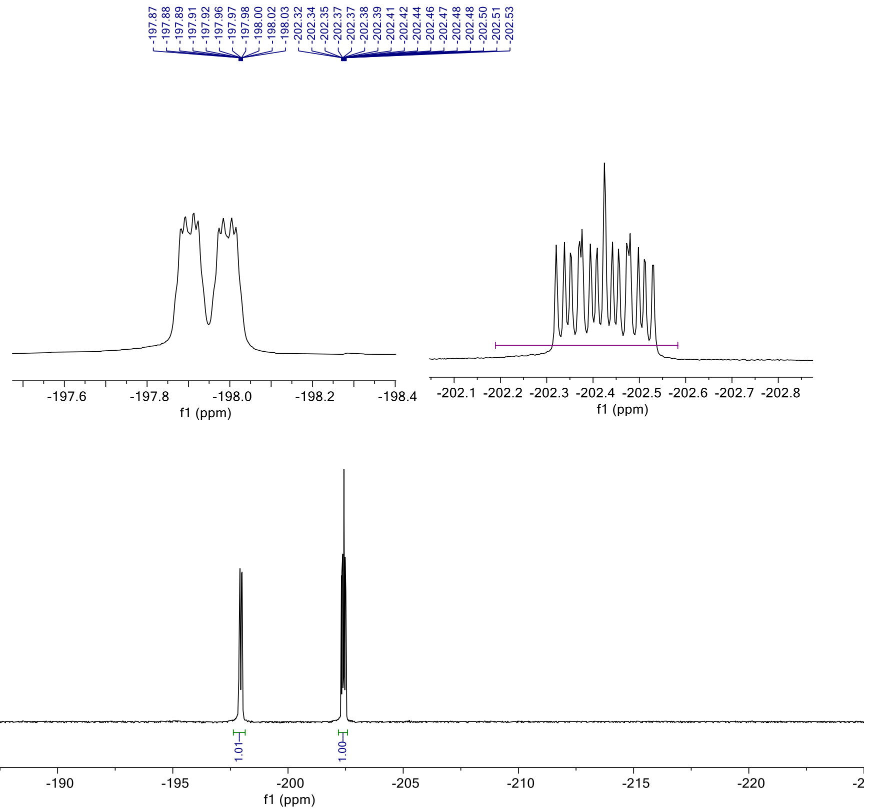

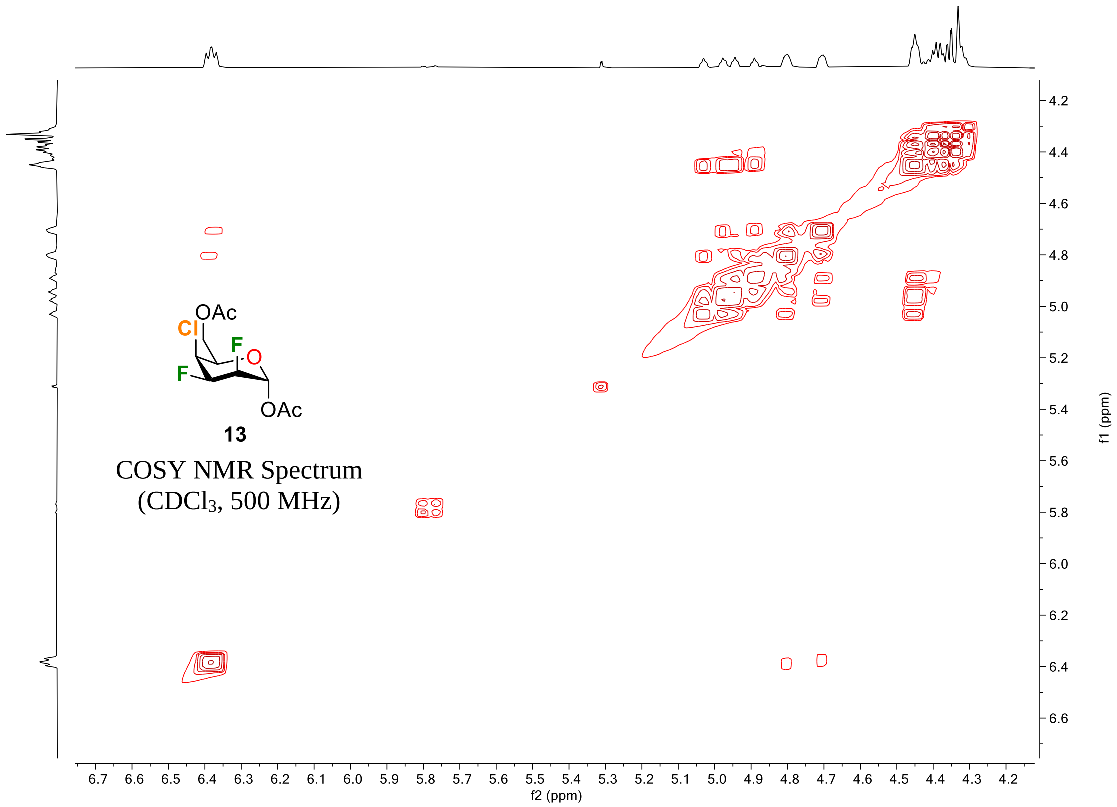

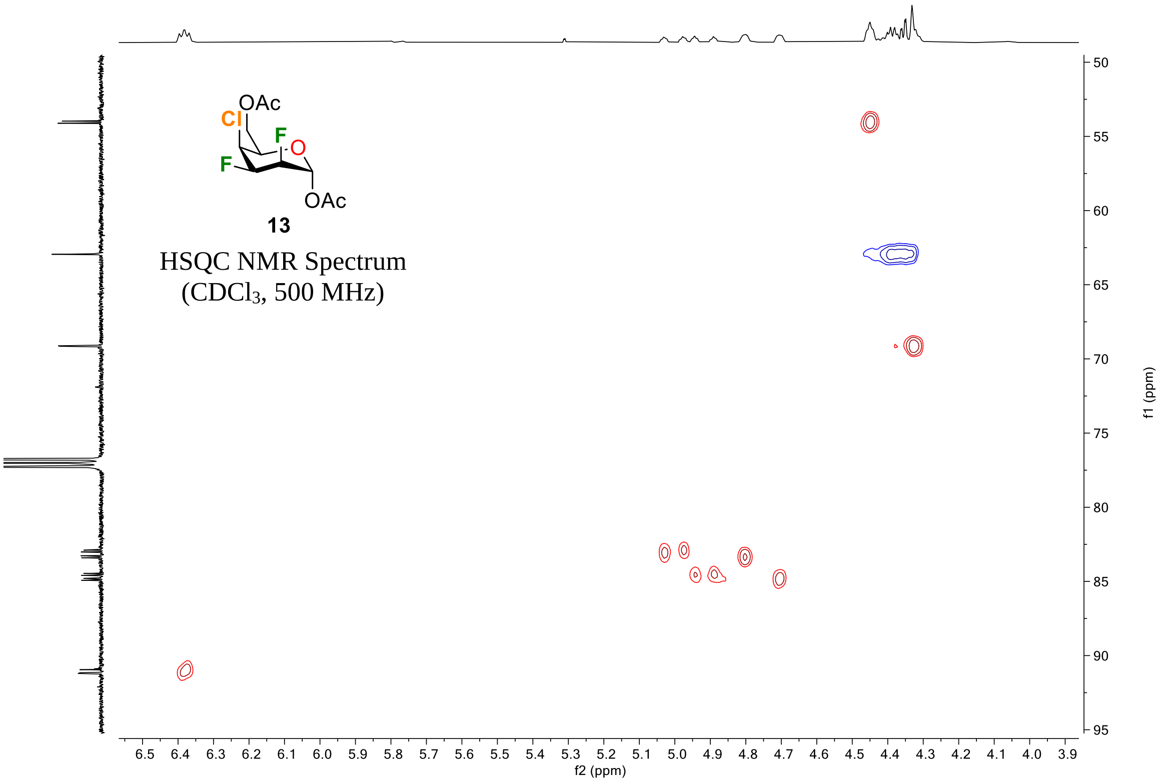
OAc
Cl
F
O
F
OAc
13
HSQC NMR Spectrum
(CDCl$_3$, 500 MHz)
f2 (ppm)
f1 (ppm)

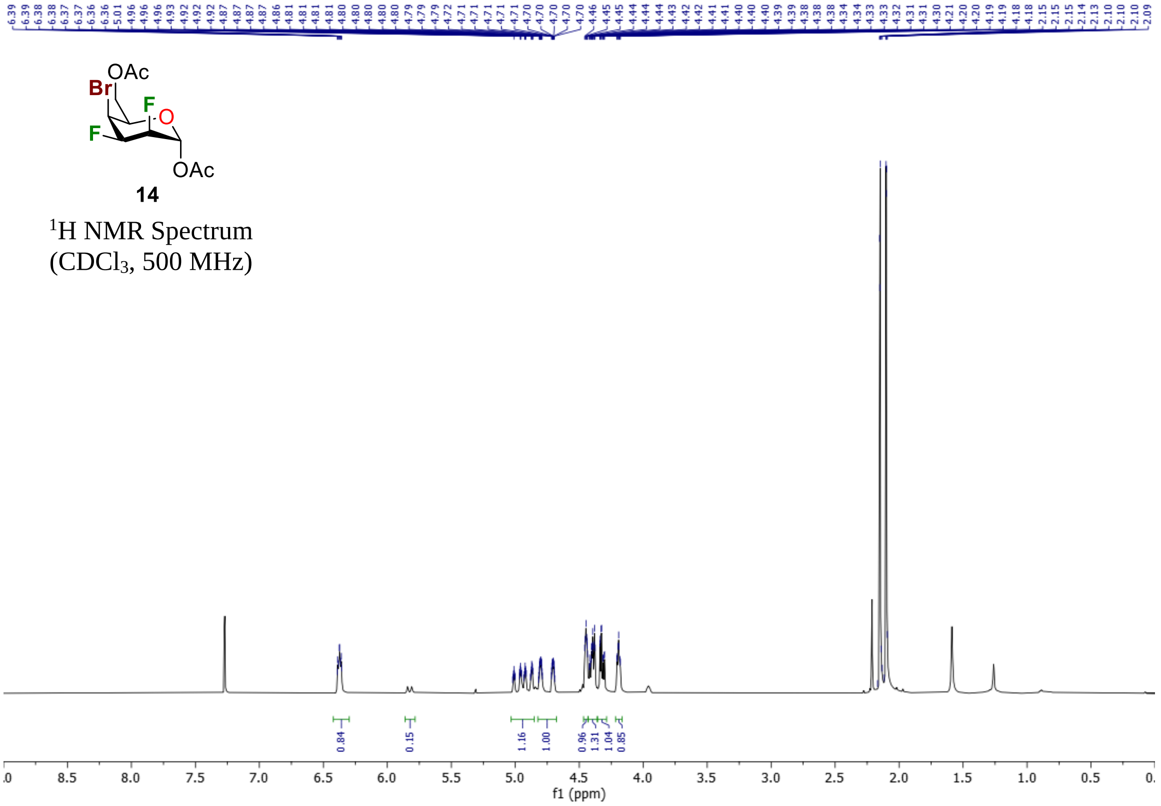
OAc
Br
F
O
F
OAc
14
^{1}H NMR Spectrum
(CDCl$_3$, 500 MHz)
f1 (ppm)

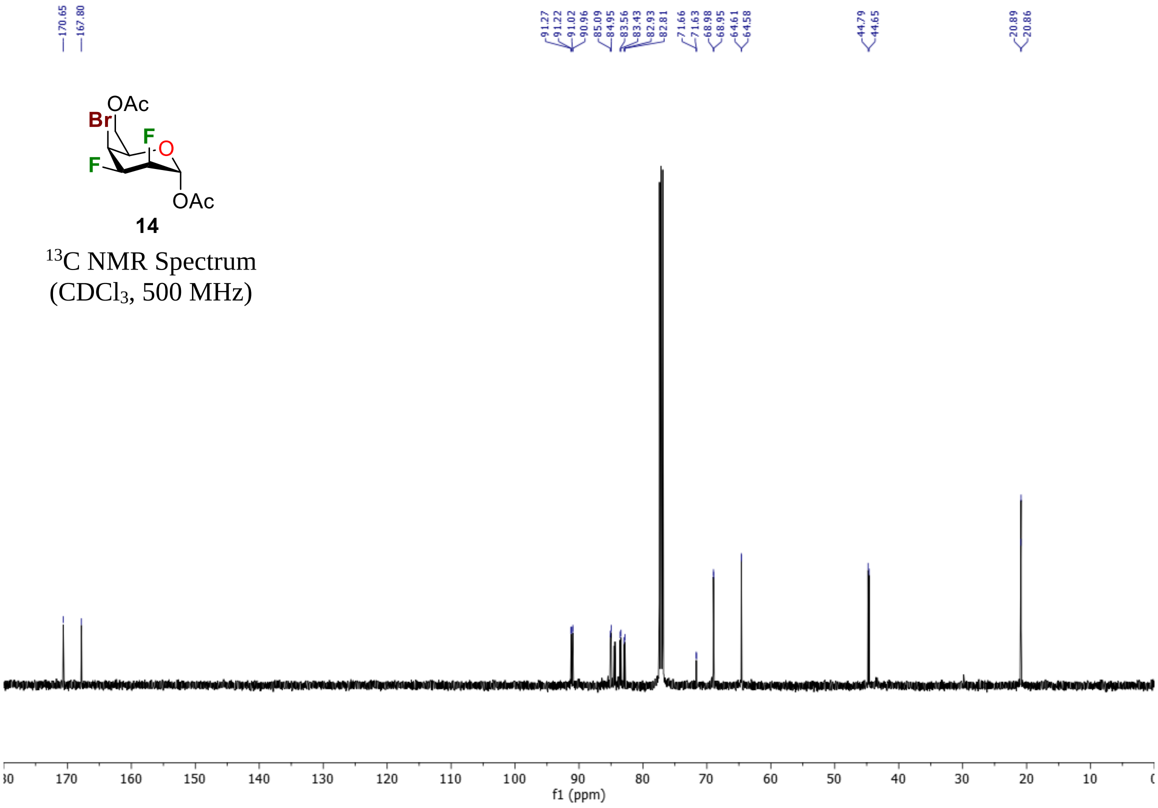
14
^{13}C NMR Spectrum
($CDCl_3$, 500 MHz)
f1 (ppm)

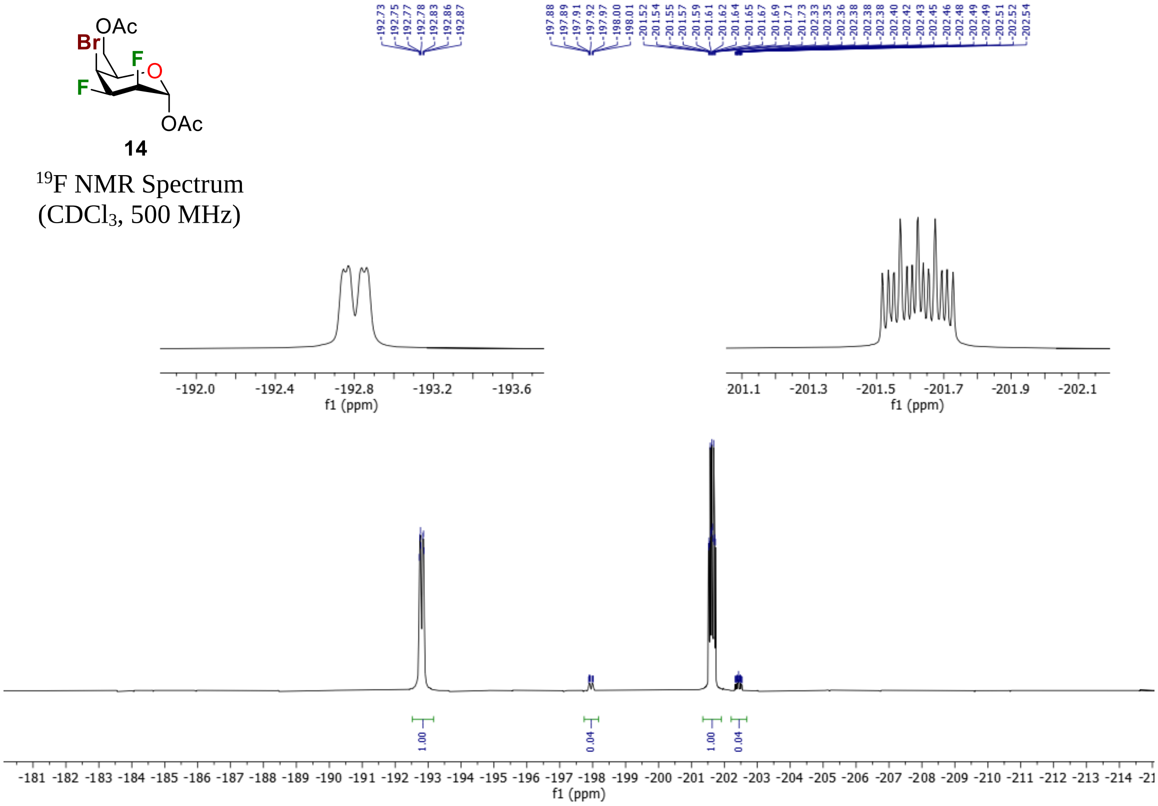
14
^{19}F NMR Spectrum
($CDCl_3$, 500 MHz)
f1 (ppm)

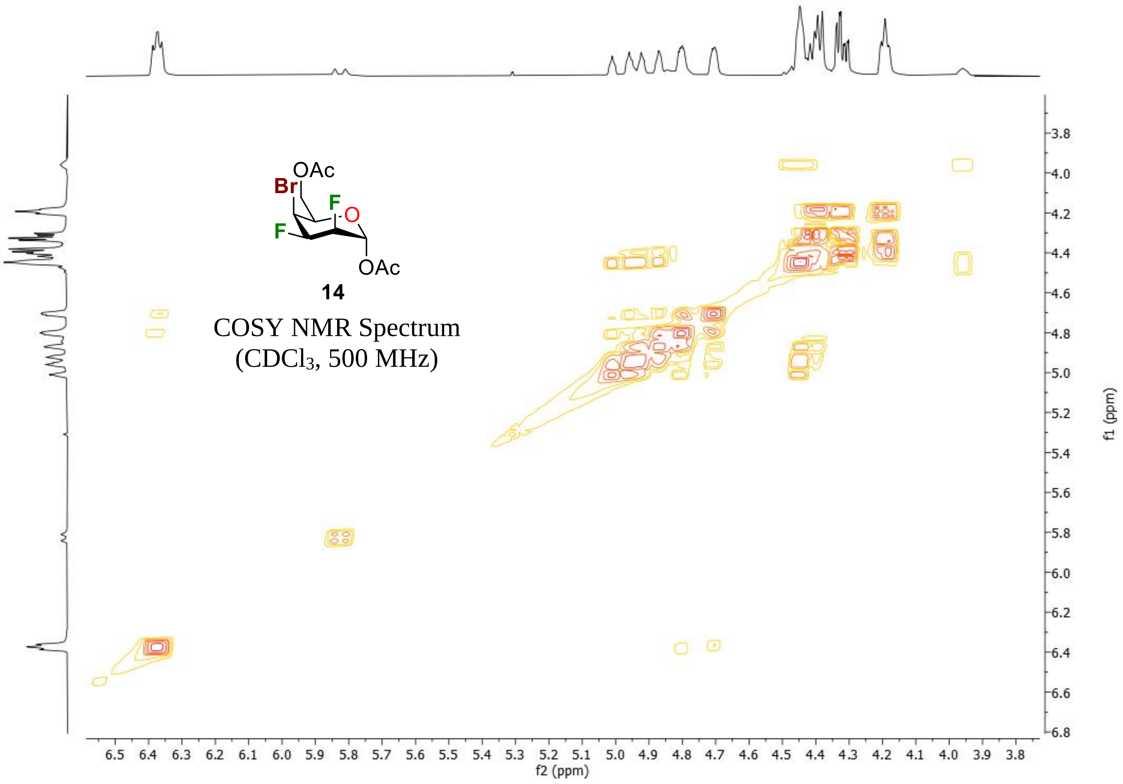
OAc
Br
F
O
F
OAc
14
COSY NMR Spectrum
(CDCl$_3$, 500 MHz)
f2 (ppm)
f1 (ppm)

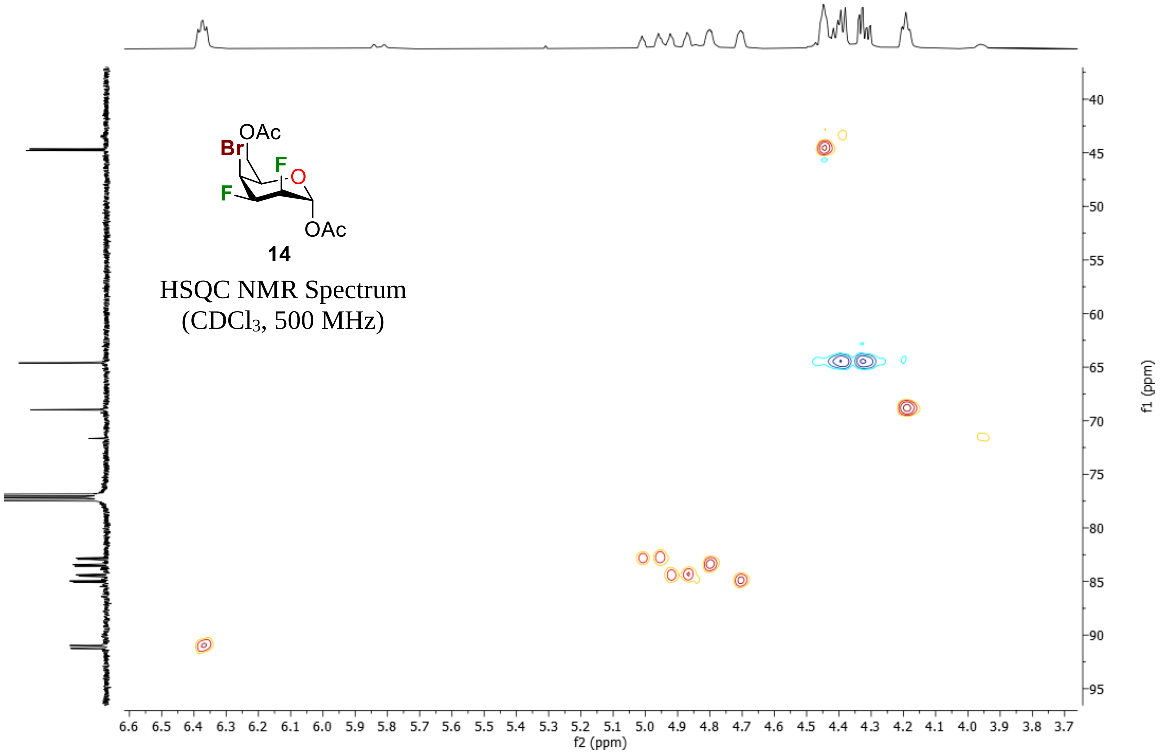
OAc
Br
F
O
F
OAc
14
HSQC NMR Spectrum
(CDCl$_3$, 500 MHz)
f2 (ppm)
f1 (ppm)

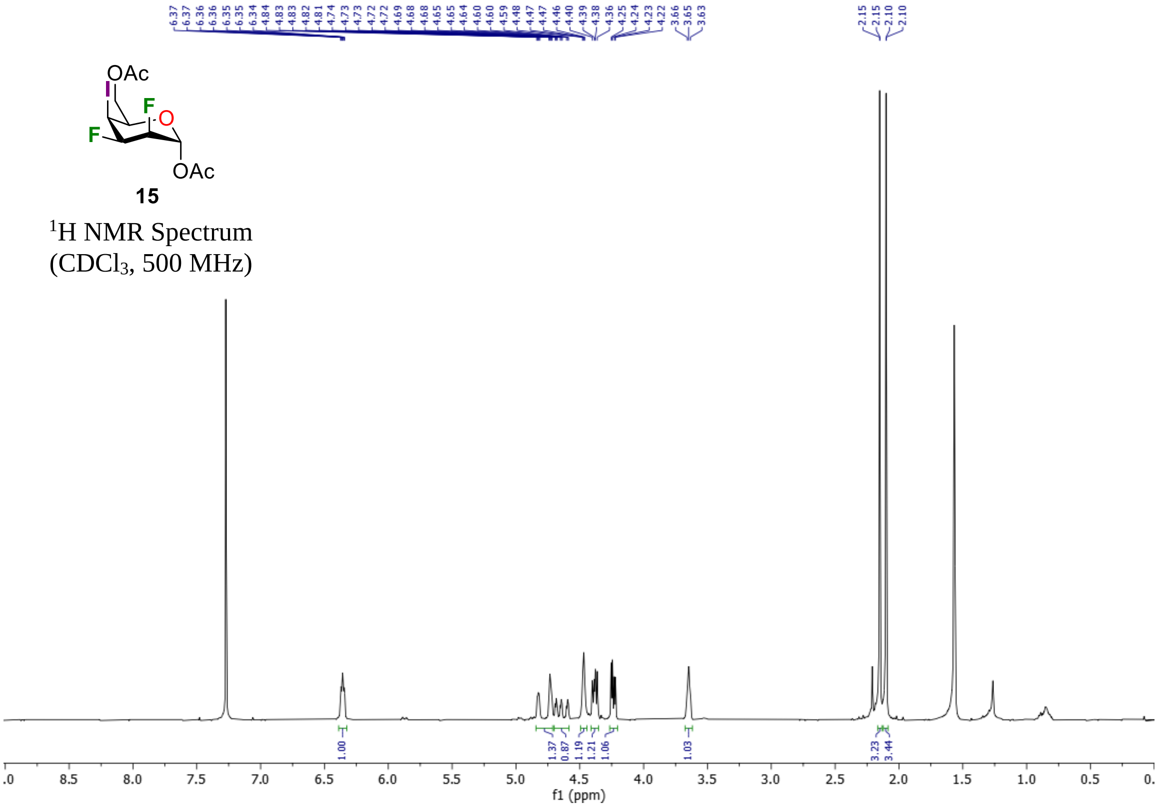

OAc
I
F
F
O
OAc
15
1H NMR Spectrum
(CDCl3, 500 MHz)
f1 (ppm)

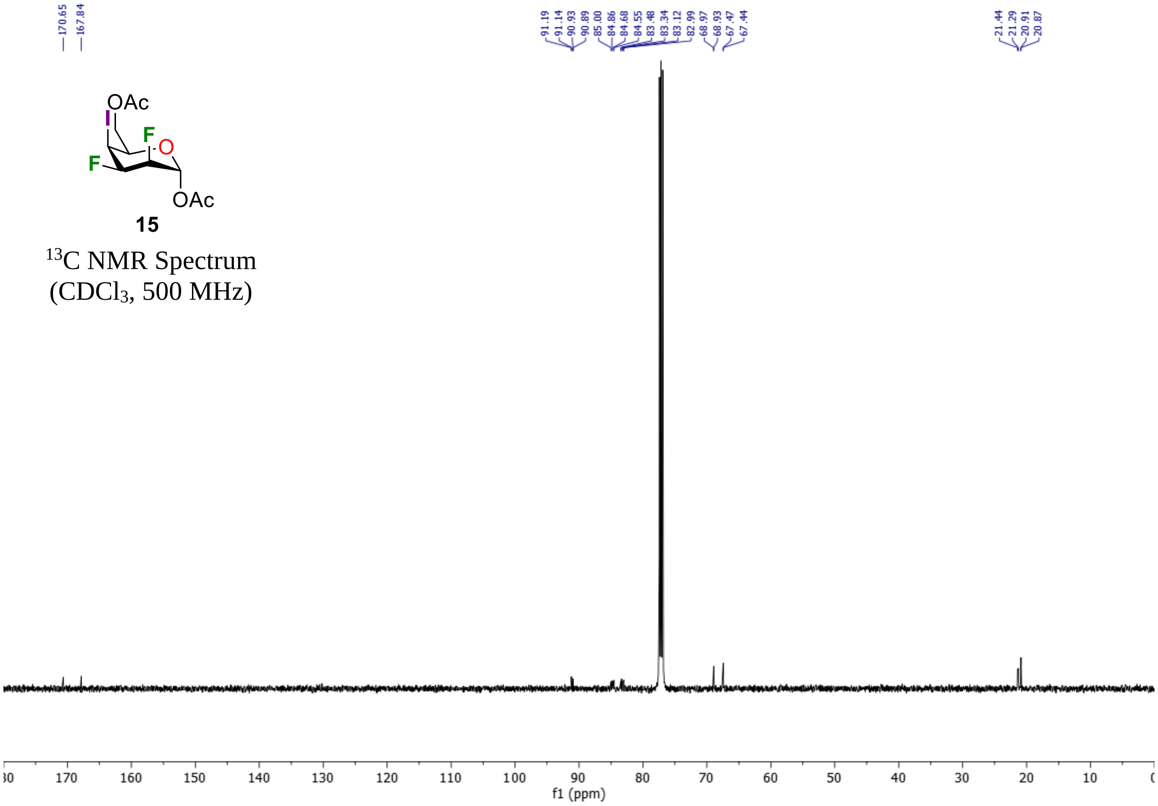

OAc
I
F
F
O
OAc
15
13C NMR Spectrum
(CDCl3, 500 MHz)
f1 (ppm)

15

^{19}F NMR Spectrum
($CDCl_3$, 500 MHz)

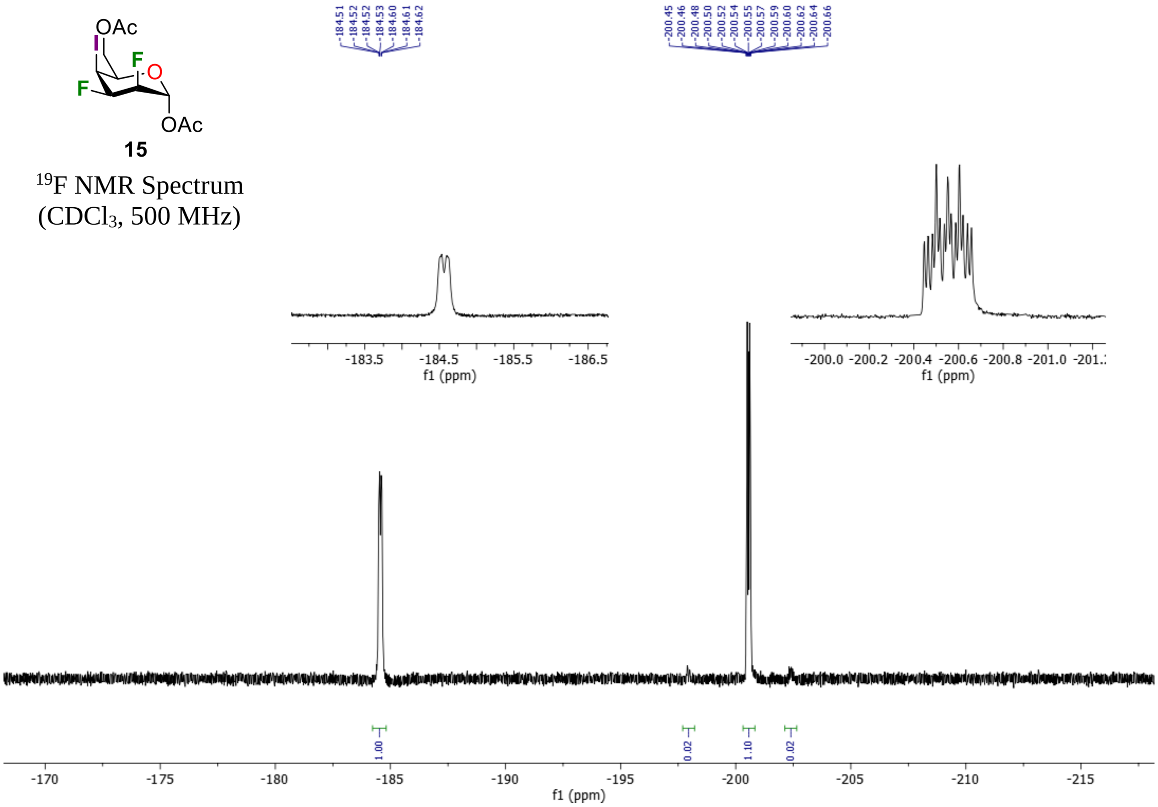

15

COSY NMR Spectrum
($CDCl_3$, 500 MHz)

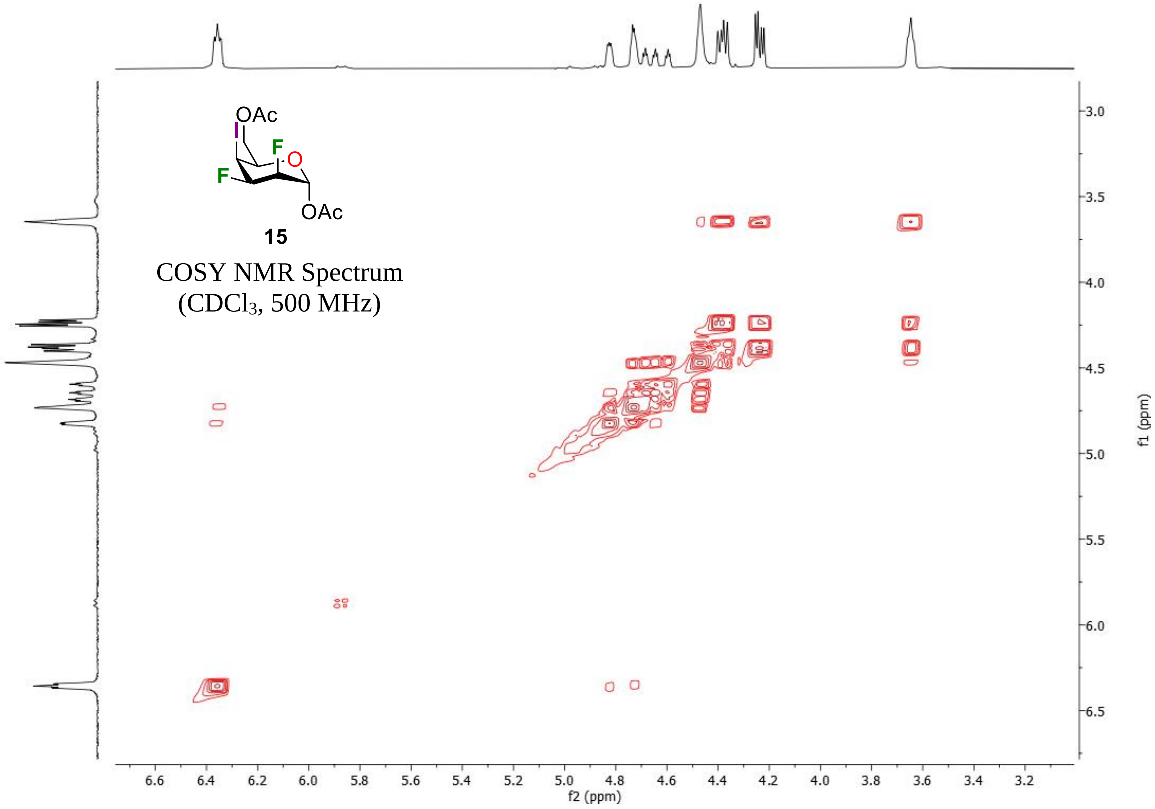

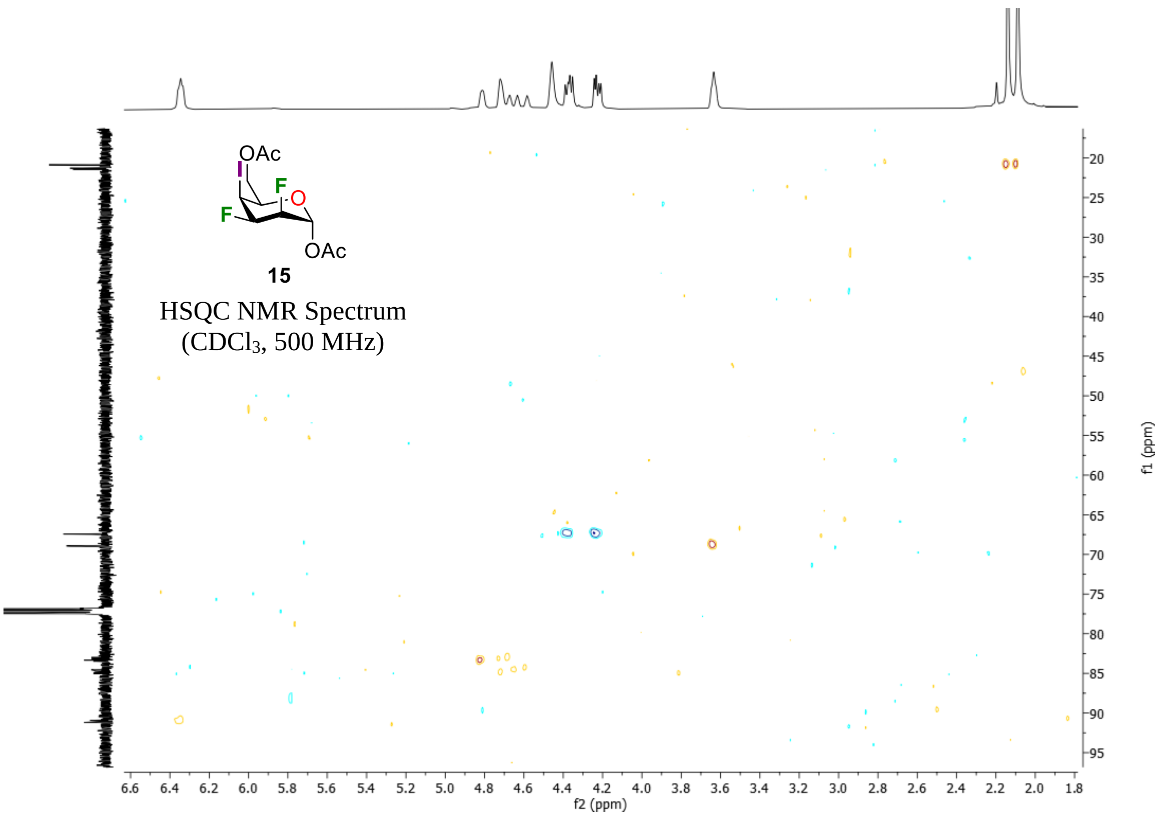

OAc
I
F
O
F
OAc
15
HSQC NMR Spectrum
(CDCl3, 500 MHz)
f1 (ppm)
f2 (ppm)

VII. References

[1] Denavit, V.; Lainé, D.; St-Gelais, J.; Johnson, P. A.; Giguère, D. *Nat. Commun.* **2018**, *9*, 4721. doi:10.1038/s41467-018-06901-y

[2] Lessard, O.; Lainé, D.; Fecteau, C.-É.; Johnson, P. A.; Giguère, D. *Org. Chem. Front.* **2022**, *9*, 6566–6572. doi:10.1039/D2QO01433E

[3] Gaussian 16, Revision B.01, M. J. Frisch, G. W. Trucks, H. B. Schlegel, G. E. Scuseria, M. A. Robb, J. R. Cheeseman, G. Scalmani, V. Barone, G. A. Petersson, H. Nakatsuji, X. Li, M. Caricato, A. V. Marenich, J. Bloino, B. G. Janesko, R. Gomperts, B. Mennucci, H. P. Hratchian, J. V. Ortiz, A. F. Izmaylov, J. L. Sonnenberg, D. Williams-Young, F. Ding, F. Lipparini, F. Egidi, J. Goings, B. Peng, A. Petrone, T. Henderson, D. Ranasinghe, V. G. Zakrzewski, J. Gao, N. Rega, G. Zheng, W. Liang, M. Hada, M. Ehara, K. Toyota, R. Fukuda, J. Hasegawa, M. Ishida, T. Nakajima, Y. Honda, O. Kitao, H. Nakai, T. Vreven, K. Throssell, J. A. Montgomery, Jr., J. E. Peralta, F. Ogliaro, M. J. Bearpark, J. J. Heyd, E. N. Brothers, K. N. Kudin, V. N. Staroverov, T. A. Keith, R. Kobayashi, J. Normand, K. Raghavachari, A. P. Rendell, J. C. Burant, S. S. Iyengar, J. Tomasi, M. Cossi, J. M. Millam, M. Klene, C. Adamo, R. Cammi, J. W. Ochterski, R. L. Martin, K. Morokuma, O. Farkas, J. B. Foresman, and D. J. Fox Gaussian 16, Revision B.01, Gaussian, Inc., Wallingford CT, 2016.

[4] Becke, A. D. *J. Chem. Phys.* **1993**, *98*, 5648–5652. doi:10.1063/1.464913

[5] Lee, C.; Yang, W. Parr, R. G. *Phys. Rev. B* **1988**, *37*, 785–789. doi:10.1103/physrevb.37.785

[6] Yanai, T.; Tew, D.; Handy, N. *Chem. Phys. Lett.* **2004**, *393*, 51–57. doi:10.1016/j.cplett.2004.06.011

[7] Weigend, F.; Ahlrichs, R. *Phys. Chem. Chem. Phys.* **2005**, *7*, 3297–3305. doi:10.1039/b508541a

[8] a) Grimme, S.; Antony, J.; Ehrlich, S.; Krieg, H. *J. Chem. Phys*. **2010**, ***132***, 154104. doi:10.1063/1.3382344

[9] Grimme, S.; Ehrlich, S.; Goerigk, L. *J. Comp. Chem*. **2011**, ***32***, 1456–1465. doi:10.1002/jcc.21759

[10] Becke, A. D.; Johnson, E. R. *J. Chem. Phys.* **2006**, *124,* 014104. doi:10.1063/1.2139668

[11] Tomasi, J.; Mennucci, B.; Cammi, R. *Chem. Rev.* **2005**, *105*, 2999–3093. doi:10.1021/cr9904009.
